# Deep learning-assisted radiomics facilitates multimodal prognostication for personalized treatment strategies in low-grade glioma

**DOI:** 10.1038/s41598-023-36298-8

**Published:** 2023-06-11

**Authors:** P. Rauch, H. Stefanits, M. Aichholzer, C. Serra, D. Vorhauer, H. Wagner, P. Böhm, S. Hartl, I. Manakov, M. Sonnberger, E. Buckwar, F. Ruiz-Navarro, K. Heil, M. Glöckel, J. Oberndorfer, S. Spiegl-Kreinecker, K. Aufschnaiter-Hiessböck, S. Weis, A. Leibetseder, W. Thomae, T. Hauser, C. Auer, S. Katletz, A. Gruber, M. Gmeiner

**Affiliations:** 1 Department of Neurosurgery, Kepler University Hospital, Wagner-Jauregg Weg 15, 4020 Linz, Austria; 2 Johannes Kepler University, Altenberger Strasse 69, 4040 Linz, Austria; 3 Department of Neurosurgery, Clinical Neuroscience Center, University Hospital, University of Zurich, Zurich, Switzerland; 4 Machine Intelligence in Clinical Neuroscience (MICN) Lab, Department of Neurosurgery, Clinical Neuroscience Center, University Hospital Zurich, University of Zurich, Frauenklinikstrasse 10, 8091 Zurich, Switzerland; 5 Institute of Statistics, Johannes Kepler University, Linz, Austria; 6 ImFusion GmbH, Munich, Germany; 7 Institute of Neuroradiology, Kepler University Hospital and Johannes Kepler University, Linz, Austria; 8 Institute of Stochastics, Johannes Kepler University, Linz, Austria; 9 Institute of Pathology and Neuropathology, Kepler University Hospital and Johannes Kepler University, Linz, Austria; 10 Department of Neurology, Kepler University Hospital and Johannes Kepler University, Linz, Austria

**Keywords:** Network models, CNS cancer

## Abstract

Determining the optimal course of treatment for low grade glioma (LGG) patients is challenging and frequently reliant on subjective judgment and limited scientific evidence. Our objective was to develop a comprehensive deep learning assisted radiomics model for assessing not only overall survival in LGG, but also the likelihood of future malignancy and glioma growth velocity. Thus, we retrospectively included 349 LGG patients to develop a prediction model using clinical, anatomical, and preoperative MRI data. Before performing radiomics analysis, a U2-model for glioma segmentation was utilized to prevent bias, yielding a mean whole tumor Dice score of 0.837. Overall survival and time to malignancy were estimated using Cox proportional hazard models. In a postoperative model, we derived a C-index of 0.82 (CI 0.79–0.86) for the training cohort over 10 years and 0.74 (Cl 0.64–0.84) for the test cohort. Preoperative models showed a C-index of 0.77 (Cl 0.73–0.82) for training and 0.67 (Cl 0.57–0.80) test sets. Our findings suggest that we can reliably predict the survival of a heterogeneous population of glioma patients in both preoperative and postoperative scenarios. Further, we demonstrate the utility of radiomics in predicting biological tumor activity, such as the time to malignancy and the LGG growth rate.

## Introduction

### Background

As one of the most prevalent forms of CNS tumors, diffuse low grade gliomas (LGG, World Health Organization [WHO] grade 2 or 3) have distinct clinical outcomes and require different treatment strategies based on their unique clinicopathological characteristics^1,2^. In contrast to extraaxial or extracranial tumors, LGG diffusely infiltrate the brain parenchyma and can extend well beyond the original tumor mass detectable by standard radiological means. Although a comprehensive neuropsychological evaluation reveals abnormalities in the majority of patients at the time of diagnosis, subjective and clinical symptoms are typically subtle^3,4^. LGG are thus diagnosed at various stages, depending on the size, location, and growth kinetics of the tumor. They are regarded as initially slow-growing with an irrevocable propensity to transform malignant within 7–8 years, i.e. glioblastoma transition^5^.

Feasible total onco-functional resection of LGG within the brain is often deemed impossible due to its extent or location. Understanding tumor biology, such as tumor kinetics, infiltration patterns, malignant transformation tendencies or disease stage, is invariably required to maximize tumor resection and improve patient outcome. In fact, the heterogeneous landscape, different response to the same treatment, and resistance to standard treatment regimens render the “same treatment for all”—approach inadequate^6,7^. Modern techniques in neurosurgery, such as intraoperative awake monitoring and intraoperative MRI, have increased the average overall survival of LGG patients from 3 to 7 years upon diagnosis to 11 years or longer^5^. For optimal survival and neurocognitive outcome, the timing of surgery and subsequent adjuvant therapy is also essential. Currently, only a few clinical or molecular markers are routinely taken into account when determining which treatment options are best for the patient at each disease stage^8,9^. Unfortunately, such a subjective approach frequently introduces bias into patient management and can occasionally result in inappropriate treatment decisions. Consequently, clinicians are compelled to accept potentially avoidable collateral effects, such as the patient's early neuropsychological decline after radiation therapy, which could be delayed or avoided if more accurate information about the disease stage or future progression were available^8,10,11^. By disclosing what is currently invisible to the naked eye, radiomics show promising results in the non-invasive prediction of glioma behavior such as progression free survival or overall survival^12–14^. Objective, quantitative parameters that actually reflect the individual genetic tumor landscape, can assist in accurate personalized prediction models of LGG behavior.

With respect to accessible patient data, clinicians are largely required to make treatment decisions in two scenarios: preoperative and postoperative. In the preoperative setting, only limited variables pertinent to any potential tumor behavior are available, such as demographical information, cognitive state, tumor size, anatomical extent, and radiographic features such as contrast enhancement. In a postoperative setting, however, this dataset can be supplemented by data such as molecular characteristics, type of surgery, or adjuvant therapy. This study aimed to improve the applicability and efficacy of current radiomics models of overall survival for patients with low and intermediate grade glioma. In addition, as a preliminary step toward glioma behavior prediction, we developed a multiparametric models for the time to malignancy and tumor growth rate.

## Results

### Clinical characteristics

Patient characteristics in the patient cohort are shown in Supplementary Table S1. Mean follow up was 5.9 years (21 days–21.7 years). The patients were divided into a training (n = 216) and a testing cohort (n = 72).

### Segmentation performance

A U^2^ convolutional neural network was scripted for automated deep learning-based glioma segmentation. The resulting pre-trained model obtained a Dice score of 0.837 on a held-out validation set (consisting of 5% of the entire BraTS data). Five patients had to be excluded based on faulty segmentation results at this stage.

For each of the MRI sequences (T1 with and without contrast, T2 and FLAIR) pyradiomics features were calculated separately, totalling 428 features per patient. The extracted features were divided into seven groups: (1) first-order statistics: *n* = 18; (2) shape and size features: *n* = 14; (3) grey-level co-occurrence matrix (GLCM) features: *n* = 24; and (4) grey-level-run-length matrix (GLRLM) features: n = 16; (5): grey-level-size-zone matrix (GLSZM) features: n = 16; (6): neighboring-grey-tone-difference matrix (NGTDM) features: n = 5; (7): grey-level-dependence matrix (GLDM) features: n = 14. Following feature reduction steps n = 17 remained.

### Prediction model performance (overall survival)

Cox proportional hazards models with different sets of predictors were fit to the data. Age was centered at 45 years. Summary tables for these models are presented in Supplementary Tables S2–S7.

**Figure 1:**
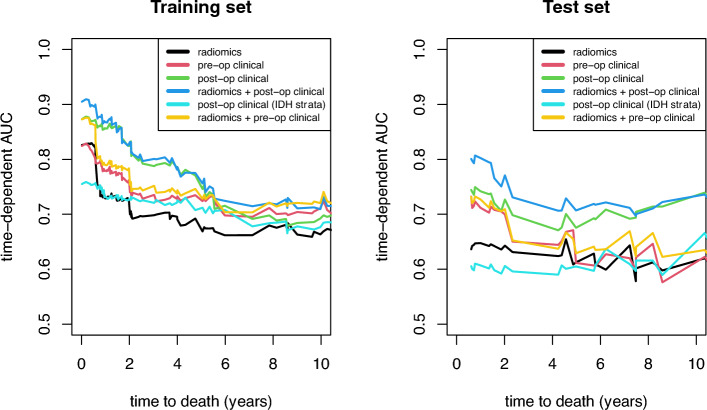
Performance evaluation: Time-dependent AUC (tAUC) for overall survival prediction with final models. In both the training set and the test set, the optimal model comprised postoperative information and radiomic characteristics.

Figure 1 depicts the progression of the time-dependent AUC value versus the time to death for several models. The radiomics model outperforms the pre-op clinical model for the randomly generated test set (n = 216). Adding post-op clinical data resulted in an additional boost in performance. These characteristics also apply to the test set (n = 72). Four years after surgery, the tAUC values for the test set are nearly 0.7 for post-operative clinical data but only slightly higher than 0.6 for radiomics data. However, the model that extends the post-op clinical model by including radiomics data achieved the highest predictive quality. For both the test and training sets, this model outperforms the post-op clinical model. For the test set, the tAUC value for predicting death 1 year after surgery was around 0.8. In later years, the value remained around 0.7. Stratifying the post-op clinical model by IDH mutation did not achieve the desired effect; the predictive quality decreased considerably when compared to the unstratified model.

The C-index in Table 1 is a more general evaluation metric. Its findings were consistent with the previous findings regarding tAUC. The model using both radiomics and post-op data achieved a C-index of 0.82 (CI 0.79–0.86) for the training set, which significantly outperformed the model using only radiomics data: 0.72; (CI 0.68–0.77). Pre-op and post-op clinical models were lined up in the middle. The c-indices for the test set showed similar patterns.

**Table 1:** Predictive model performance metrics: C-index and bootstrapped 95% CI for training and test set; B = 1000.

	Training set	Test set
Radiomics	0.72 (0.68, 0.77)	0.63 (0.52, 0.72)
Pre-op clinical	0.76 (0.71, 0.80)	0.66 (0.56, 0.77)
Post-op clinical	0.81 (0.77, 0.85)	0.71 (0.60, 0.83)
Radiomics + post-op clinical	0.82 (0.79, 0.86)	0.74 (0.63, 0.85)
Post-op clinical (IDH strata)	0.72 (0.67, 0.78)	0.60 (0.51, 0.72)
Radiomics + pre-op clinical	0.77 (0.73, 0.82)	0.67 (0.56, 0.78)

### Time to malignancy

Supplementary Table S10 provides parameter estimates based on preoperative clinical data. Three distinct scenarios are analyzed:

- *Presence of malignancy at diagnosis (a)* The probability of malignant tumors at diagnosis significantly increases with age and tumors located on the left or median side. Factors such as sex, tumor morphology (diffuse/expansive), and cortical architecture showed no significant effects.
- *Time to malignancy between diagnosis and surgery (b)* Age played a significant role in the risk of malignancy, with a higher age correlating to an increased likelihood. Additionally, tumor morphology was a notable factor, with the covariate 'expansive morphology' reducing the hazard.
- *Time to malignancy following surgery (c)* This case showed a high level of uncertainty as none of the Cox model covariates, including age, demonstrated strong statistical significance. This may be attributed to the exclusion of patients with malignant tumors before surgery, leaving only 145 patients in the analysis. The impact of cortical architecture was found to be borderline significant, with a *p* value of 0.0619.

### Tumor growth velocity

**Figure 2:**
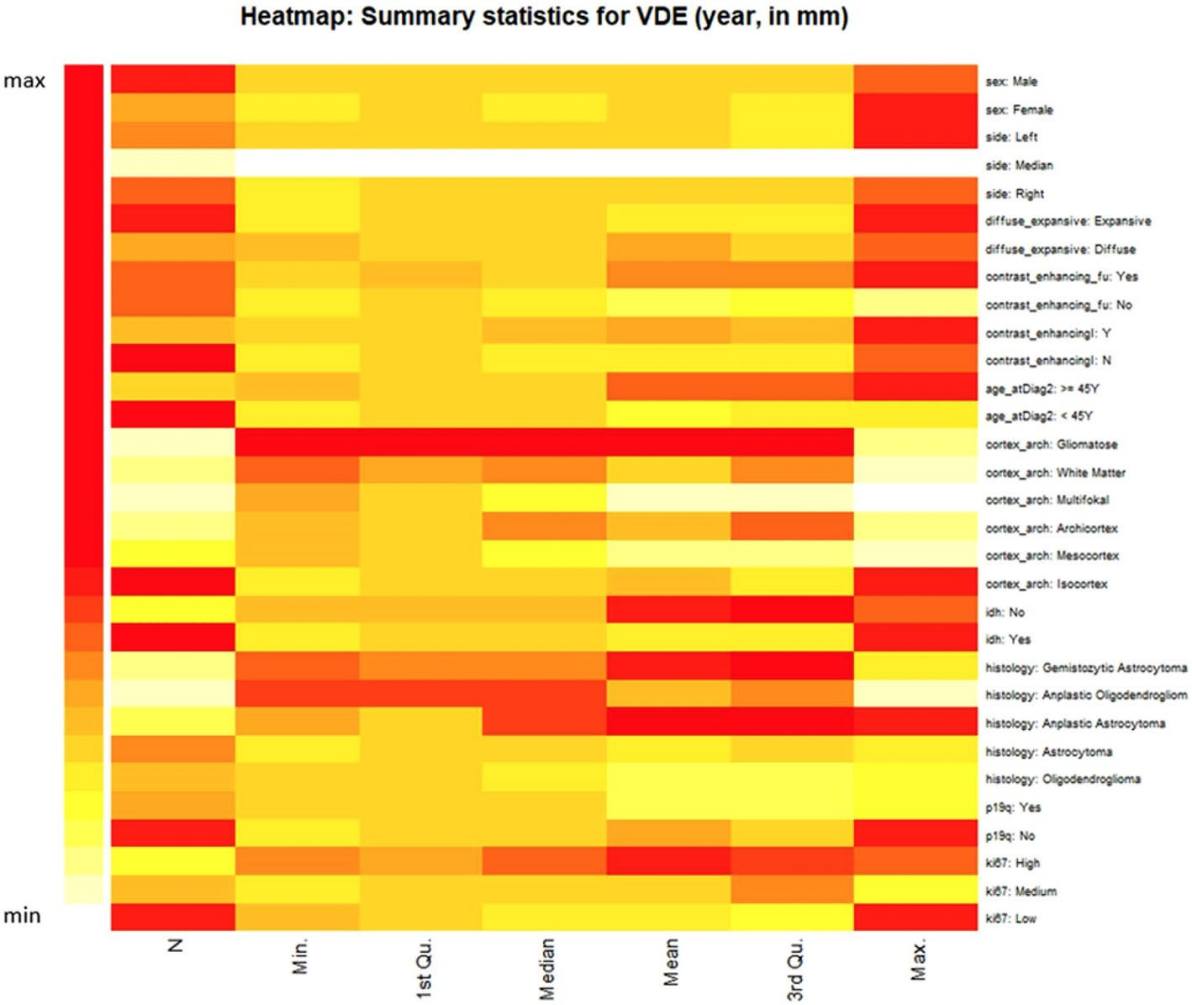
Glioma growth velocity: Heatmap depicts influential clinical factors on mean tumor diameter per year. Anaplastic histology and gliomatosis expectedly show the highest VDE. Cortex architecture and Ki-67 proliferation index show similar differences in mean VDE.

Both relative tumor growth velocity and mean tumor diameter (calculation as proposed by Pallud et al.^15^) were calculated. Gliomatosis (diffuse tumor involvement of three or more lobes) was significantly associated with mean relative tumor growth per day. The heat map depicts a general trend in significance for factors that have been shown to contribute to overall survival, such as IDH status, multifocality, and cell proliferation index (see Fig. 2). After performing the previously described feature reduction steps, radiomic features were also significantly associated with relative tumor growth velocity per day.

## Discussion

**Figure 3:**
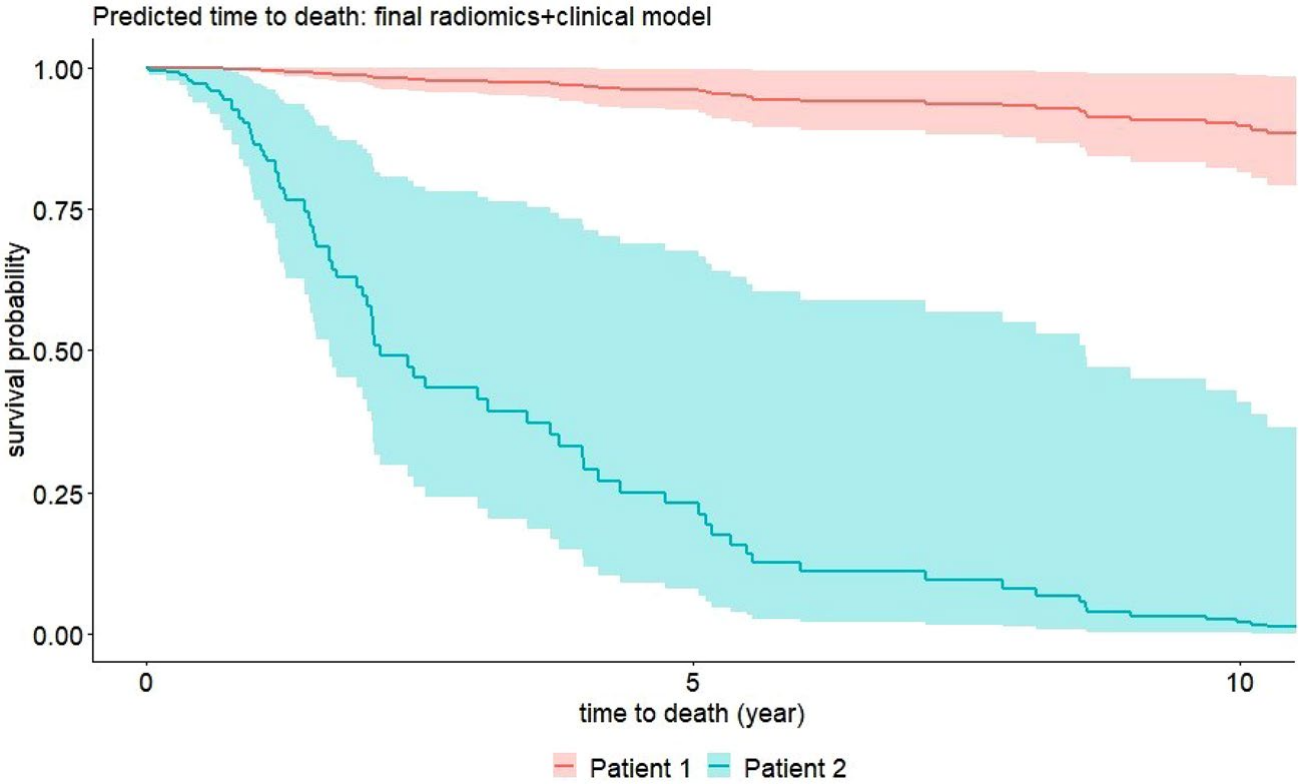
Prediction of Survival: Predicted survival curves incl. 95% intervals based on the post-op clinical model for two patients with IDH mut, Ki67 = low. Patient 1 has Oligodendroglioma, and radiomic features at median values, whereas Patient 2 has Anaplastic Astrocytoma, and radiomic features at the 75% quantile values. All other covariates have baseline values for both patients.

Translating molecular signaling pathways to heterogeneous macroscopic cellular behavior, and ultimately tumor kinetics and infiltration patterns, has proven difficult for biologists and physicians^16–21^. In this paper, we demonstrate that anatomical phenotyping of gliomas in conjunction with deep learning-based tumor segmentation and radiomics analysis can reliably predict the forthcoming behavior of gliomas (see Fig. 3).

Predictive capacities are not limited merely to projections of overall survival; rather, we demonstrate that dynamic tumor behavior, such as the propensity for malignant transformation or expected tumor growth velocity, may also be computed.

One of the major challenges for radiomic models remains the heterogeneous imaging data used to train the segmentation algorithms. Different imaging techniques, imaging artifacts, and scan quality of the source MRIs produce significant error rates in segmentation processes, which, in addition to being time consuming, is a significant reason why automated tumor segmentation and radiomic analysis are not yet routinely performed in the clinical setting^22^. As a result, we attempted to develop a time- efficient deep learning model capable of segmenting images from various scanners in order to make our model as clinically applicable as possible. This was accomplished by pretraining the U2- model on a publicly available data set from the 2021 BraTS challenge and then fine-tuning it on 89 patient sequences from our cohort.

Despite the fact that the image quality and field strength of the included MRI were very heterogeneous, we only had to exclude five patients due to faulty segmentations. This method obtained a Dice score of 0.837, which is comparable to the top-performing state-of-the-art glioma segmentation models^23–25^.

In an effort to aid in the appropriate characterization and to identify aggressive or indolent glioma variants at any given stage of the tumor or treatment stage, we constructed pre- and postoperative prediction models. Naturally, we consider radiomics as a potent ally for this endeavor, as evidenced by the fact that the inclusion of radiomic elements enhanced all of our predictive models.

Our approach demonstrates a reliable prediction of 10-year overall survival, achieving a concordance index (C-index) of 0.82 (95% CI 0.79–0.86) for the training cohort and 0.74 (95% CI 0.64–0.84) for the test cohort in a postoperative context. In a preoperative setting, the model yields a C-index of 0.77 (95% CI 0.73–0.82) for the training cohort and 0.67 (95% CI 0.57–0.80) for the test cohort. To the best of our knowledge, our study presents the most effective overall survival prognostic model for a real life LGG-cohort to date, with a particular emphasis on extended survival analysis. Notably, our results provide a comprehensive 10-year overall survival evaluation, which bears significant relevance for LGG patients who often exhibit extended survival periods. This distinction underscores the potential clinical applicability and broader implications of our findings within the field of LGG management.

Li et al.^26^ demonstrated significant results in predicting overall survival by distinguishing high- and low-risk patients and were able to validate their findings in a prospective cohort. However, they relied on manual segmentation of the tumor region of interest and disregarded anatomical information. Based on manual ROI-delineation, Yan et al.^27^ achieved a C-index for OS using a model for both high and low grade gliomas of 0.806 (95% CI 0.740–0.872) in the training cohort and 0.735 (95% CI 0.621–0.872) in the validation cohort. In a research study involving cohorts from the Chinese Glioma Genome Atlas (CGGA) and The Cancer Imaging Archive (TCIA), Qian et al. presented a C-index of 0.70 for overall survival prediction in the validation cohort by employing a nomogram that integrated radiomic features^28^.

Similar to our findings, Choi et al.^29^ were able to demonstrate an improvement in Area under the Curve (iAUC) in an LGG cohort when clinical data alone were compared to radiomics data in addition to clinical data (0.627 vs. 0.709, respectively). Radiomics has also demonstrated its utility by successfully differentiating between distinct molecular profiles, such as IDH mutation versus wild type, 1p/19q co-deletion^26,30,31^. In a separate study, Liu et al.^32^ reported a C-index of 0.815 in a validation cohort for progression-free survival assessment. Furthermore, they identified positive correlations between radiomic characteristics and biological activities, such as cellular proliferation and the development of blood vessels.

Time to malignancy and tumor growth velocity are equally important to a physician as overall survival, not only because they are inextricably linked, but also because they are a significant aid in determining the level of aggressiveness of the underlying tumor entity. In our study, radiomic parameters had a significant impact on both, thereby complementing clinical parameters.

**Figure 4:**
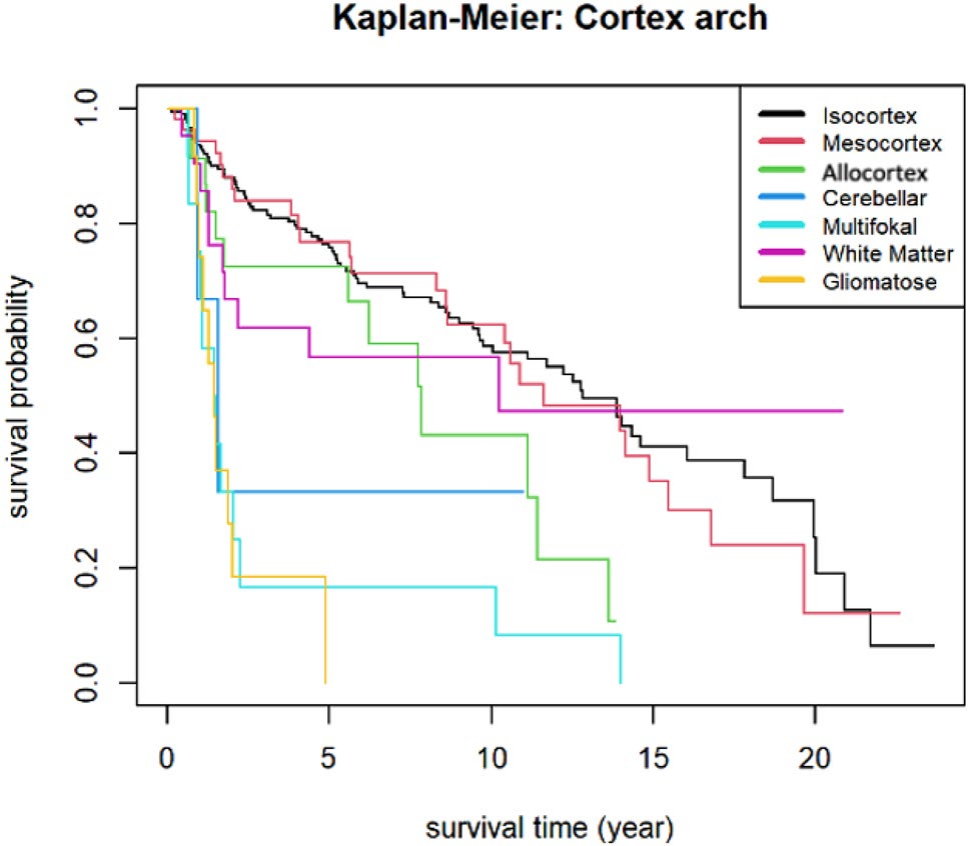
Association between cortical architecture and overall survival: A Kaplan–Meier graph illustrates the association between phylogeny of tumor origin and overall survival in our cohort. Tumors with mesocortical topology have the most beneficial survival whereas gliomatosis or multifocality have the worst prognosis.

Many reports have shown a strong correlation between phylo- ontogenetical origin, histocompartimentalization and glioma behavior^16,18,19,21^. We therefore considered it essential to include precise topological and phylogenetic data. In our series we were able to demonstrate significant differences in the natural history of the disease based on the architecture of the affected cortex (see Fig. 4), which were reflected not only in the Kaplan–Meier curves but also in the final OS prediction models.

Furthermore, topological and phylogenetic information appear to influence tumor growth velocity and time to malignant transformation. Zhou et al.^33^, built radiomic models integrating approximate anatomical information via VASARI features^34^ but could not find a significant effect of tumor location with overall survival. A small number of patients and a lack of exact phylogenetic data likely contributed to the inability to detect any significant topological effects. Similar studies identified relationships between mutation status and lobar involvement when anatomical labeling by VASARI characteristics was implemented in their separate prognostic models^31,35^.

It has to be noted that the degree of uncertainty for some results was still rather considerable. This was the result of the limited number of study participants and the random separation of the data into training and test sets. In this analysis, the results for the c-indices were linked with wide confidence ranges of 95 percent, particularly for the test set. Also, the standard errors for several coefficient estimations were quite high.

This was especially evident for the regression analysis of the interval-censored “time to malignancy”. As a result, effects that may very well be present could not be demonstrated with sufficient certainty. Including additional patients in the dataset would have in all probability enhanced the quality of the analysis. Seventeen percent of the patients in our group had to be excluded, primarily due to incomplete clinical data or missing MRI sequences. Sparsity is a challenge for model performance because large disparities within a single feature class with few parameters can have a significant impact on prediction output.

In future studies, we intend to enhance the efficacy of our technique by including patients from additional institutions, by employing advanced MRI imaging and more sophisticated molecular genetic information. We did not include DWI or perfusion-based imaging because they have not been widely used in clinical practice for a long time, potentially reducing the number of patients who could have been included.

Further research is warranted to externally validate the prognostic models, ensuring their generalizability and robustness in different contexts.

In conclusion, we built a MRI radiomics model based on a deep learning segmentation algorithm to predict OS and glioma behavior in a heterogeneous patient group both before and after surgery. Moreover, we were able to successfully apply our deep learning strategy to glioma segmentation on a realistic data set with few restrictions and without subjective rater bias. The correct interpretation of the glioma stage or its inherent level of aggressiveness will be of major importance in the pursuit of an optimal onco-functional balance in glioma patients.

## Methods

Ethics board approval was obtained prior to data acquisition from the local ethics committee (JKU-Ethikkommission, EK-2021-1042). All patients or their legal representatives gave their legal informed consent and the study conducted in accordance with the Declaration of Helsinki. The results are reported in accordance with the STROBE statement.

### Source population

We established a retrospective data base using a CALUMMA data management system (RISC Software, Austria). The data were obtained from a consecutive patient cohort of 349 patients with a newly diagnosed WHO grade 2 or 3 glioma admitted to the Kepler University Hospital in Linz between 1999 and 2022. The surgeries were all performed as part of the clinical routine with preoperative neuroradiological evaluation and postoperative follow up MRI.

The eligibility criteria comprised: (1) first diagnosis of a histologically verified grade 2 or 3 supratentorial glioma; (2) availability of preoperative MRI scans including T1 with and without contrast enhancement, T2 and FLAIR; (3) no pretreatment or previous cranial surgery. Patients were excluded if (1) the automated deep learning segmentation showed erroneous results or (2) if artifacts or low image quality of the transversal slices of the beforementioned MRI sequence were likely to interfere with an acceptable radiomics analysis. To fit the final models, 288 patients were included.

The demographic, clinical, and histological information is presented in Supplementary Table 1.

For brain imaging of the source population, 43 distinct MRI scanners (0.4–3 Tesla) from multiple institutions following various clinical protocols were used. Tissue samples were analyzed at the Institute of Pathology and Microbiology 2 and the Department of Neurosurgery, Unit for Theoretical Neurosurgery, at the Kepler University Hospital Linz.

The minimally required MRI-protocol for the neural network-based tumor segmentation consisted of: T_1_ pre-and post-contrast-weighted images, T_2_-weighted images and fluid-attenuated inversion recovery (FLAIR).

### Study design

**Figure 5:**
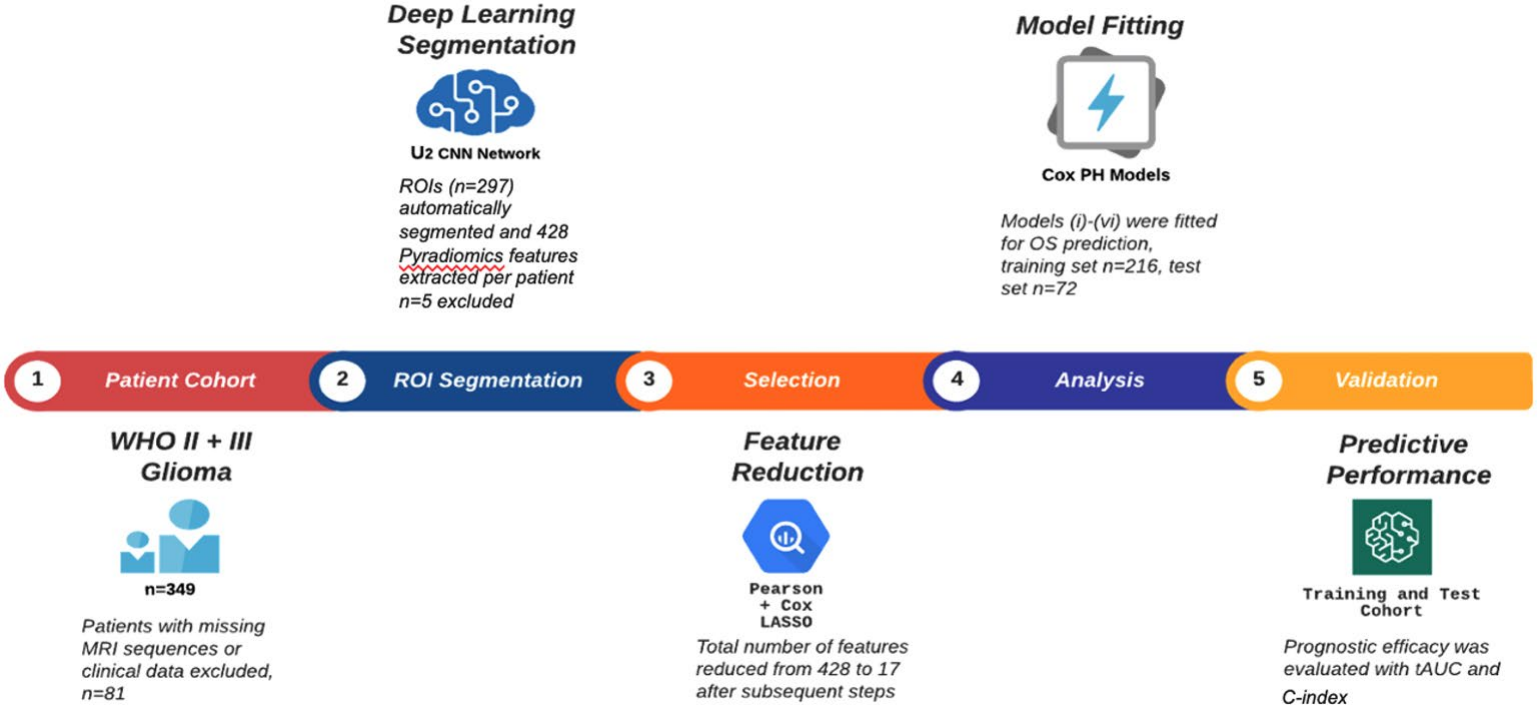
Radiomics Workflow: The entire radiomics pipeline was divided into five successive steps which are depicted here.

As depicted in Fig. 5, this study design involves five phases of consecutive steps up to the final prediction model:

1. Cohort analysis

Preoperative MRI scans were qualitatively analyzed according to an adapted topological and phylogenetic tumor extension protocol provided by Akeret et al.^16^. Anatomical structures that were only rated displaced or edematous, but not invaded, were classified as non-affected. In some cases, the consultation of the post-resection MRI study was helpful in this decision.

According to its limits and the visible displacement of neighboring structures, the tumor shape was further characterized as either expansive or diffuse. Additionally, contrast enhancement was graded as "yes/no" and a tumor was categorized as "malignant" if the contrast enhancement was present alongside an increase in rCBV. New contrast enhancement with or without rCBV increase was likewise evaluated as "malignant transformation" in follow-up scans. Image analysis was performed by two expert investigators (P.R. and M.A.) with a third (M.S.) aiding to reach consensus in the case of disagreement.

(2) Segmentation pipeline and preprocessing

**Figure 6:**
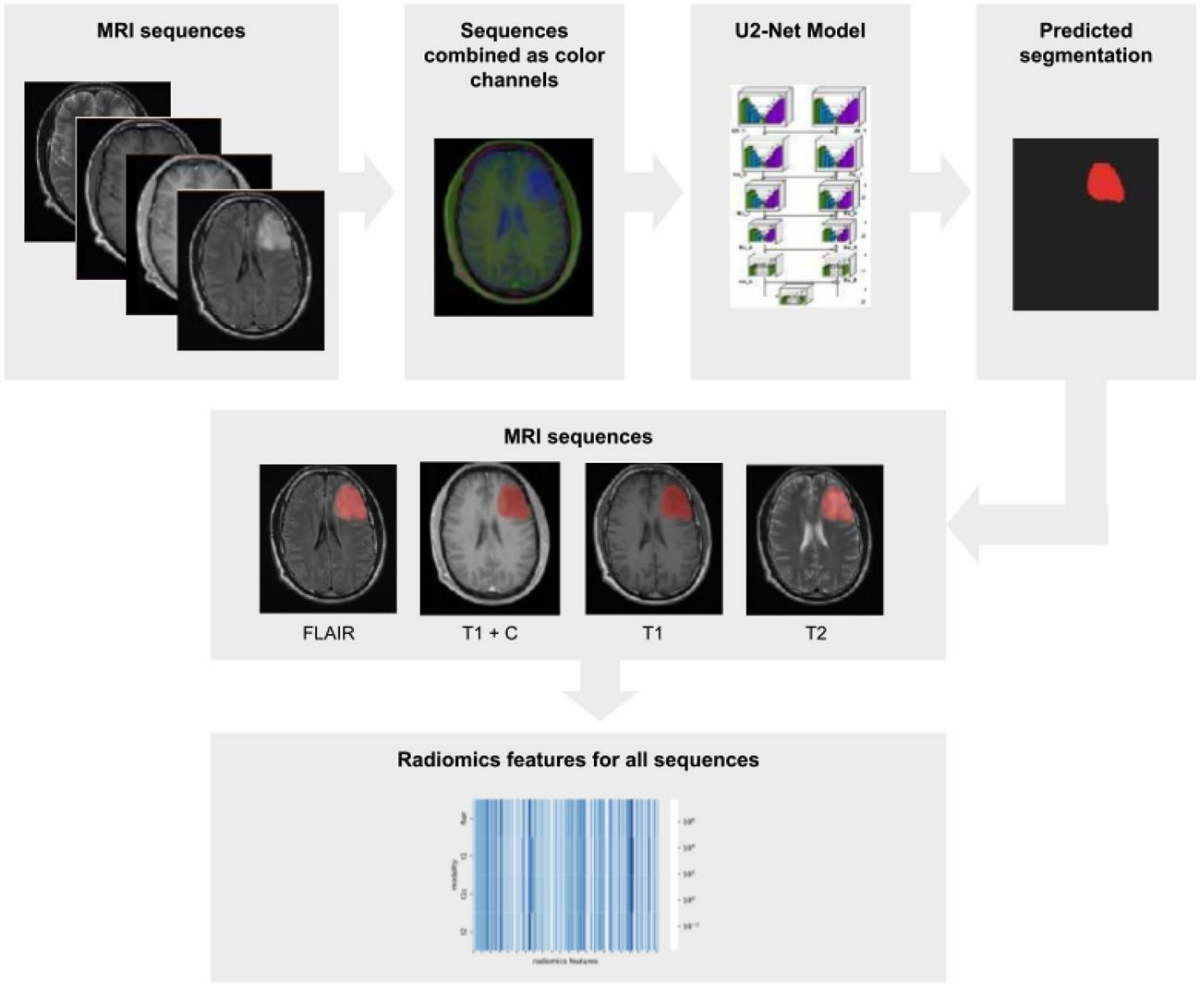
Deep-learning for automated glioma segmentation: MRI processing with U^2^-convolutional neuronal network segmentation and Radiomics feature extraction pipeline.

As a second step, we applied a deep learning-based automatic segmentation, followed by an automated feature extraction (see Fig. 6). For the radiomics study of the tumors, a segmentation mask of the relevant tumor is required. To eliminate concerns emerging from inter- and intra-rater disagreement^36^, we trained a neural network to generate a cohesive collection of tumor segmentations for the study. Therefore, 89 sets of axial cranial MRI scans were randomly chosen from our internal clinical database. Each set included T1, T1 with contrast, T2, and FLAIR scans of the same subject. T2-weighted images were labeled by radiological experts (P.R., M.A., M.S.) using the ImFusion Labels (Imfusion, Munich, Germany). Due to the scarcity of training data, the model was pre-trained on the BraTS2021 dataset^37^ before fine-tuning on the study datasets.

### Pre-training on BraTS

The preferred model in this study was the U^2^-Net^38^. Following numerous experiments, the optimal model setup consisted of four downsampling blocks and a channel count starting at 12. The network's convolutional layers consisted of a 3d convolution followed by instance normalization^39^ and ReLU activation, with the exception of the layers immediately preceding the output, which employed Sigmoid activation. As input to our model, we concatenated the T1, T1 with contrast, T2, and FLAIR modalities for each patient in the BraTS dataset^37,40,41^. We merged the necrosis, enhancing tumor, and edema classes seen in the BraTS data into a single tumor class for our purposes. Clipping the top and bottom percentiles, subtracting the mean, and dividing by the standard deviation normalized the images for model training. To increase the generalizability of the pre-trained model to our clinical dataset, random augmentations were employed during training. These consisted of random variations in image intensity with an amplitude of no more than 0.2, random rotations of the image in any direction by no more than 15 degrees, and random flipping of the image axes. We sampled 128 × 128 × 128 sub-volumes from the scans during training. We trained the model for 39 epochs (early stopping) with a batch size of 12 using an Adadelta optimizer^42^ with a learning rate of 1 and a combination of Dice and Binary Cross-Entropy losses.

### Fine tuning of clinical data

As the clinical radiological data is much more heterogeneous compared to BraTS, the distribution of voxel sizes present in our dataset was identified as a first step (see Supplementary Fig. 4). From there, we settled on a training resolution of 0.4 × 0.4 × 4.5 mm^3^ as it was roughly the 25th percentile for each dimension in our training data. Given this resolution, we fine- tuned the model on 256 × 256 × 16 sub-volumes after resampling the scans with trilinear interpolation. In addition, since the scans from the different MR sequences were not always well aligned, we used the ImFusionSuite (Imfusion, Munich, Germany) software^43^ to register all sequences to the corresponding T2-weighted scan. We initialized the registration using the center of mass of the scans and used a rigid registration using mutual information as the similarity measure. Aside from the different resolutions, the training setup was the same as in the pre-training stage. We used 80 sets for the training and kept 9 for the validation set. The final model trained for 15 epochs (early stopping) on the clinical dataset.

### Radiomic feature extractions

We obtained the tumor masks for the scans in this study using the final fine-tuned model. During inference, we applied the same preprocessing (excluding random augmentations) as during the model's training. We used a connected-components analysis to select only the main tumor volume to ensure that the radiomics features would not be biased by smaller satellites and spurious detections.

For the feature extraction, we used the widely-used python package pyradiomics^44^. We resampled the scans to a resolution of 1 × 1 × 1 and used the built-in normalization and binning capabilities of pyradiomics^44^. We set the bin width to 0.05.

Before the feature extraction the segmentation performance for each patient was evaluated and, if necessary, the patient was excluded from further analysis. We refrained from manually correcting erroneous segmentations to ensure a radiomics study devoid of subjectivity.

The detailed calculation formula for each RF is provided on the official website (https://pyradiomics.readthedocs.io/en/latest/), accessed on July 7th 2022.

Z-score standardization (subtraction of the mean and division with the standard deviation) of the radiomics features was considered during data-preprocessing.

(3) Feature reduction

**Figure 7:**
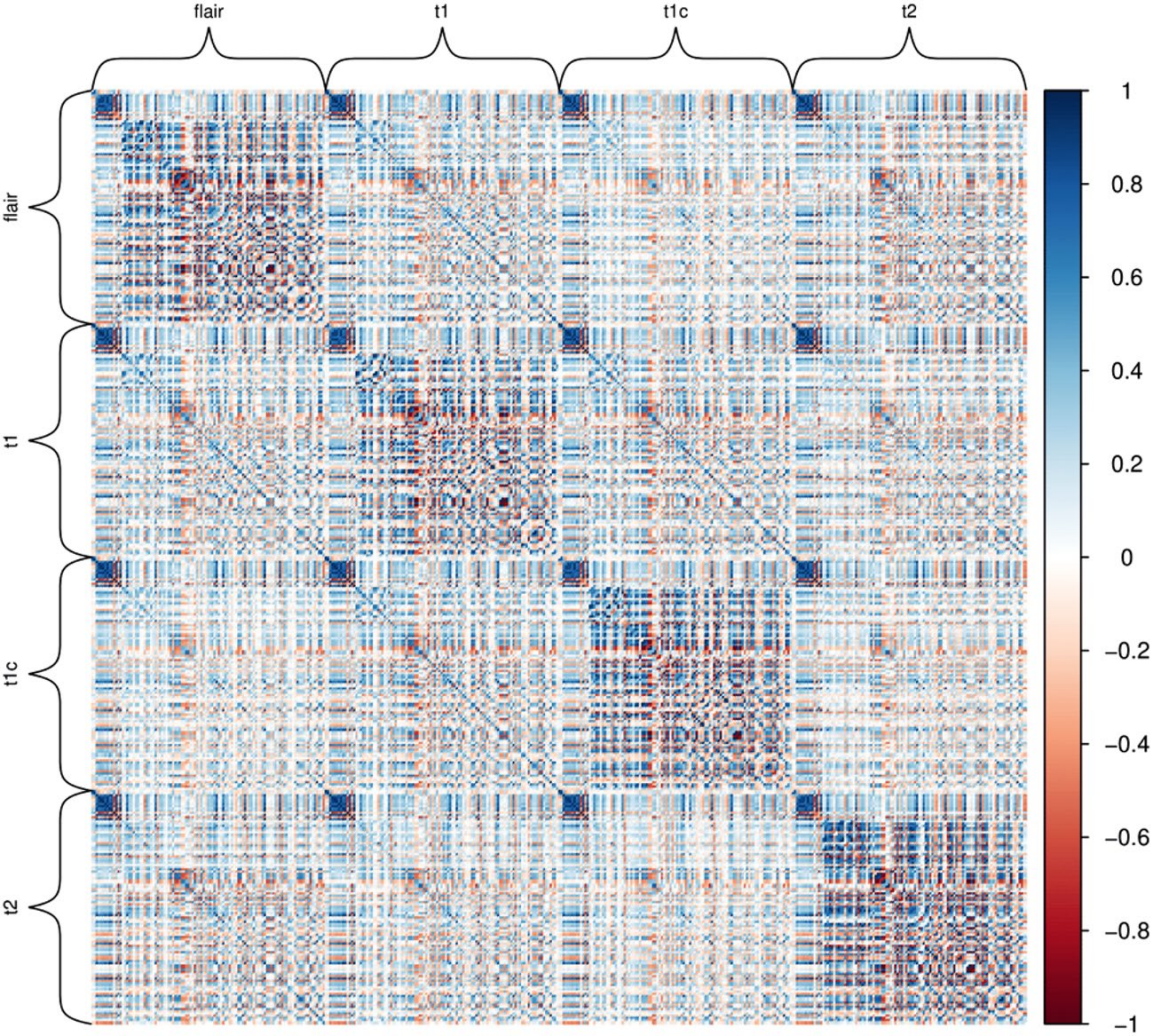
Heatmap of combined MRI sequences and radiomic features: Pearson correlation matrix for modalities FLAIR, T1, T1c and T2 (428 features). Correlations > 0.8 were disregarded to achieve a feature reduction.

To prevent overfitting in statistical models, a series of variable selection steps were taken. For the radiomic features, first, pairwise Pearson correlations were computed (see Fig. 7).

If the absolute correlation of two features was larger than 0.8, the feature with the larger mean of absolute pairwise correlation to all other features was removed (using function *findCorrelation* in R-package *caret).* Next, the data was randomly split into a training (75%) and a test (25%) set. Only the training set was used for the subsequent model building steps.

To achieve further reduction of radiomics features for modelling overall survival of patients Cox-LASSO (least absolute shrinkage and selection operator) with the optimal λ determined by k-fold cross-validation (R-package glmnet) was applied. The LASSO performs estimation of the feature effects via a constraint on the sum of their absolute values and can result in shrinking of coefficients to zero, thus achieving variable selection, see Supplementary Fig. 8. As Cox-LASSO penalizes absolute value of the regression effects it was performed using the standardized features in the training data, resulting in 17 radiomics features with non-zero coefficients.

Also, clinical variables were pre-selected. Cramers V (see Supplementary Fig. 7) was computed for all potential categorical covariates to detect highly associated nominal features (e.g. histology and 1p/19q-Co-deletion or cortex architecture and gyral involvement). Variables with high Cramers’V to others and/or an excessive number of missing values, such as PET yes/no or TERT mutation status (see Supplementary Figure S1) were excluded for building prognostic models.

The resulting number of remaining radiomics features was used as covariates for further model fits.

(4) Model Fitting

Pre-operative data consists of information that can be gathered prior to a surgical procedure, whereas post-operative data comprises variables that become accessible only after the surgery has been performed. Our radiomics predictive models were created with real-world clinical scenarios in mind, in which physicians may not have access to complete patient data throughout the disease's progression.

To characterize the two extreme scenarios that impact clinical decision making regarding appropriate management recommendations we built the following models.

- preoperative with clinical data including demographics (age, gender, etc.) and anatomical information (affected gyral architecture, lobe, side, etc.)
- preoperative with clinical data including the radiomic signature
- postoperative (including extent of resection, molecular signature, etc.)
- postoperative with stratification for IDH mutation
- postoperative including radiomic signature
- radiomic features alone without clinical data

For models (a–f) first full Cox-PH models with the appropriate subset of the variables selected in step (3) above were fitted to the training data. Then, to choose the final model stepwise selection based on the Akaike Information Criterion (AIC) (using R_function *stepAIC()* in R-package *MASS)* was performed. Note that, different from Cox-Lasso, standardization of covariates is not required in Cox-PH models, where the partial likelihood is maximized. For model (b) and (e), the full models consist of the final model covariates from models (a) and (f) and (c) and (f) respectively. An additional AIC step resulted in the final models. The Cox PH models that had been fitted were then examined further.

(5) Validation

As a final step the prognostic accuracy of the final models for patients’ survival over the next decade was determined. For each model the time-dependent AUC and the C-index were then computed using the R-function *risksetAUC()* (in R-package *risksetROC*) for the training as well as the test data. 95% confidence intervals for the C-index were determined by bootstrapping with B = 1000 bootstrap samples.

### Analysis of time to malignancy

In addition to overall survival, the time to malignancy was also evaluated. As malignancy is only assessed during visits, there are three distinct scenarios to consider:

- the tumor is malignant at diagnosis
- malignancy is diagnosed between diagnosis and surgery
- malignancy is diagnosed after surgery,

Initially, we employed a logit model with the response variable "malignancy at initial diagnosis, "solely utilizing the information accessible at the time of the first diagnosis (a). For patients with non-malignant tumors at diagnosis, a Cox proportional hazards (Cox-PH) model was constructed for the time to malignancy using only preoperative information (b). Additionally, for the subset of patients whose tumors were not malignant at surgery—but later progressed to malignancy—, a Cox-PH model was developed for the time to malignancy, incorporating both preoperative and postoperative information. As malignancy can only be diagnosed during visits, the data on time to malignancy is subject to interval censoring, in addition to right censoring for cases (b) and (c). For further details, please refer to Supplementary Table S9.

### Tumor growth velocity

For the investigation of tumor growth velocity, a simple linear model was employed. Until chemo or radiation therapy was administered, we manually segmented all available T2 or FLAIR images for each patient using the ImFusion Labels tool (ImFusion, Munich, Germany, 2022) to determine tumor volumes (1–41 pre-treatment MRI-protocols per patient, ø 13.02). Due to the inability of our convolutional neural network to segment postoperative scans consistently, we manually segmented MRI scans for volumetricanalysis. Mean tumor diameter (MTD) was calculated according to a formula proposed by Pallud et al. (MTD = (2 · V)^1/3^)^15^. As described earlier, cox lasso, which shrinks coefficients towards 0, was used for feature selection and the results were then used for the final model.

The following criteria were taken into consideration and consequently excluded:

1. Intervals between surgeries.
2. Intervals with MTD decrease greater than 10 percent or 2 mm due to resolving edema.
3. Patients with a complete observation duration of less than 30 days prior to start of chemo- or radiation therapy (because small measurement inaccuracies had an extreme impact on the extrapolation to a full year; e.g. extreme growth rate.

## Supplementary Information

Supplementary Information.

## Data Availability

The authors confirm that the data supporting the findings of this study are available within the article and its supplementary material. Patient data overview is provided in the supplementary material. Due to the fact that they contain information that could compromise the privacy of research participants, MRI data are not available to the public, but stored on local servers at the Kepler University Clinic, Linz. If required, they are available from the corresponding author, (H.S) upon reasonable request. The use of dynamic reporting guarantees full reproducibility of the results.

## Code availability

The statistical code that was used to develop the radiomic and prognostic models in the manuscript are available at https://github.com/PRauch1/Natural-History-LGG-R-Code.git. The code for the framework, preprocessing, and segmentation pipeline is part of Imfusion's proprietary framework and is therefore not available to the public. If required, the corresponding author will answer any emerging questions upon reasonable request.
