# Supplementary material for: Deep learning-assisted radiomics facilitates multimodal prognostication for personalized treatment strategies in low-grade glioma
Source: Sci Rep. 2023 Jun 11;13:9494. doi: 10.1038/s41598-023-36298-8 (PMC10258197; doi:10.1038/s41598-023-36298-8)
Supplement: Supplementary file 1 — Supplementary Information. [file 41598_2023_36298_MOESM1_ESM.pdf]

Supplementary Material

Deep Learning-Assisted Radiomics Facilitates Multimodal Prognostication for Personalized Treatment Strategies in Low-Grade Glioma

P. Rauch MD[1,2], H. Stefanits MD PhD[1*], M. Aichholzer MD[1], C. Serra MD[2,6], D. Vorhauer[9], H. Wagner PhD[9], P. Böhm[1], S. Hartl[1], I. Manakov PhD[5], M. Sonnberger MD[3], E. Buckwar[7], F. Ruiz-Navarro MD[1], K. Heil MD[1], M. Glöckel MD[1], J. Oberndorfer MD[1], S. Spiegl-Kreinecker PhD[1], K. Aufschnaiter-Hiessböck MD[1], S. Weis MD PhD[4], A. Leibetseder MD[8], W. Thomae MD[1], T. Hauser MD[1], C. Auer MD[1], S. Katletz[8], A. Gruber MD PhD[1], M. Gmeiner MD PhD[1]

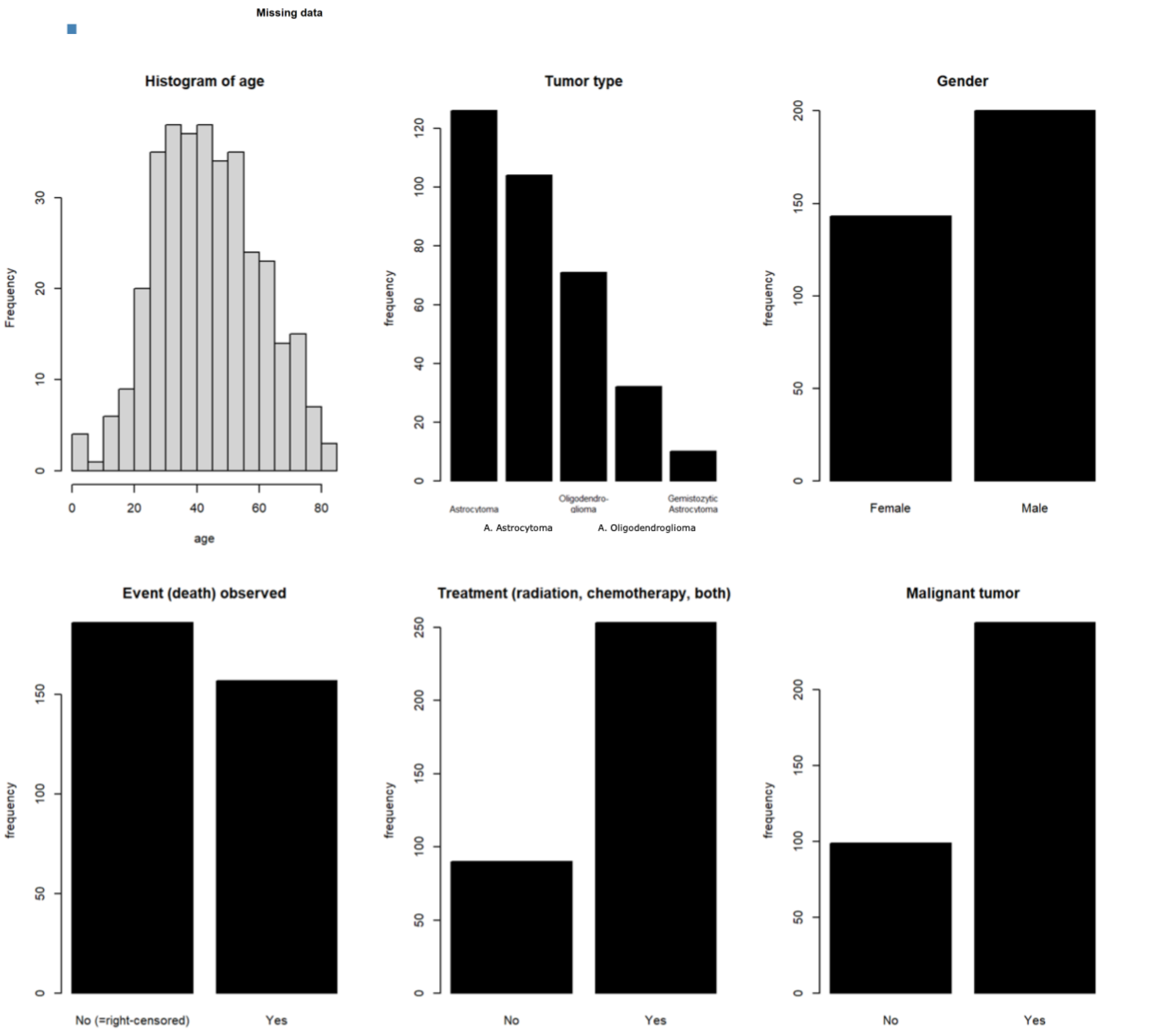

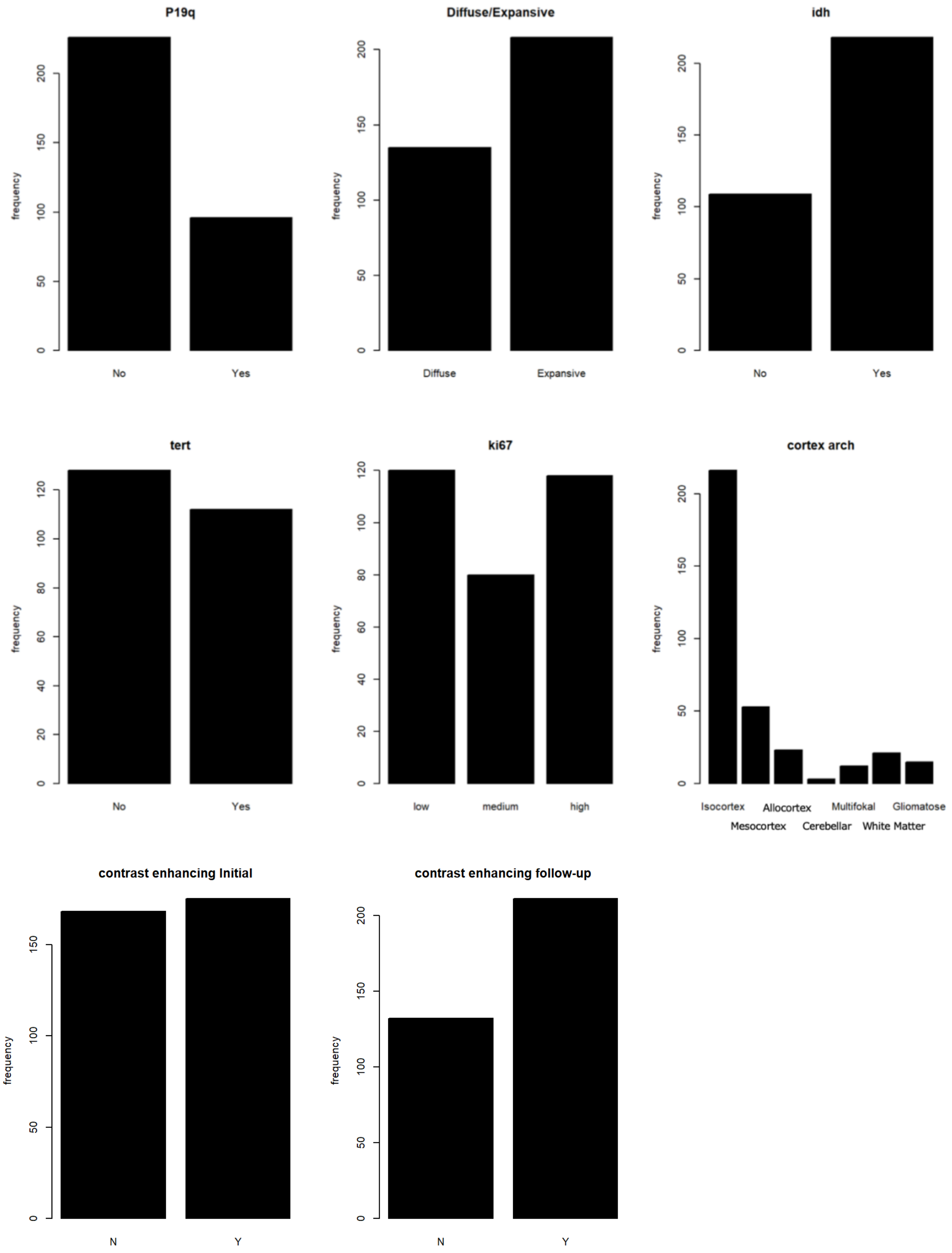

Supplementary Figure 1: Descriptive overview of relevant clinical parameters in our patient cohort

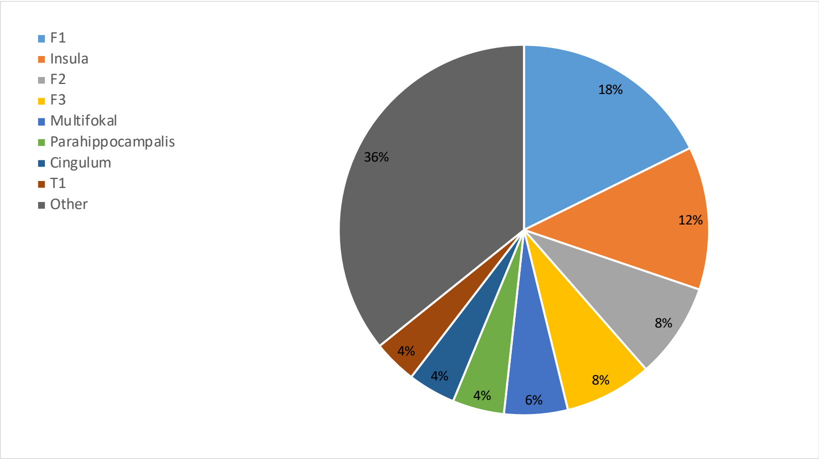

Supplementary Figure 2: A graphical representation of tumor localization on a gyral scale. Only patients used to construct the final cox models are represented in this image. F1= superior frontal gyrus, F2=middle frontal gyrus, F3=inferior frontal gyrus, T1=superior temporal gyrus

Supplementary Figure 3: Kaplan-Meier Curves for overall survival for different groups

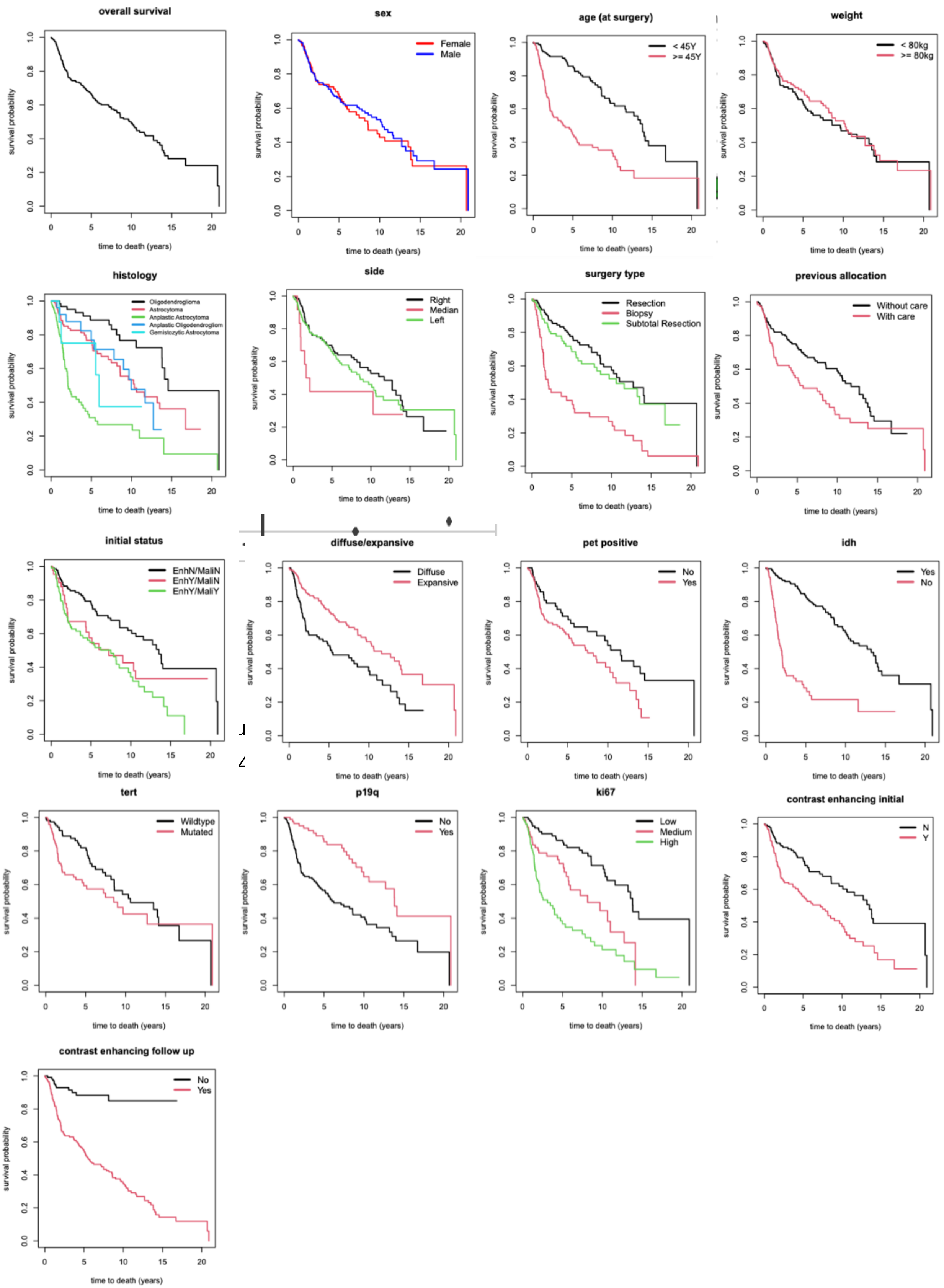

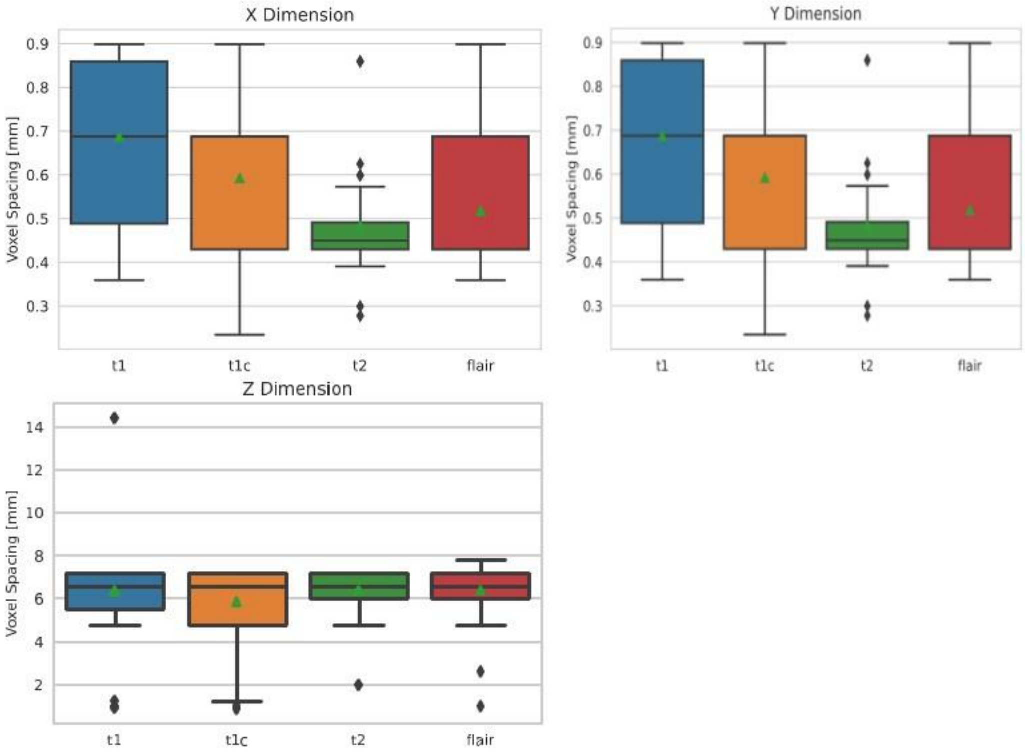

Supplementary Figure 4: Voxel Spacing distribution for CNN segmentation. We identified the distribution of voxel sizes present in our dataset in an x,y,z-axis due to heterogeneous MRI data. We chose a training resolution of 0.4 x 0.4 x 4.5 mm3 because it was approximately the 25th percentile for each dimension in our training data.

Supplementary Figure 5: Illustration of a Pearson correlation matrix for modality T1 (107 features). Online definitions are provided for the shown pyradiomics acronyms.

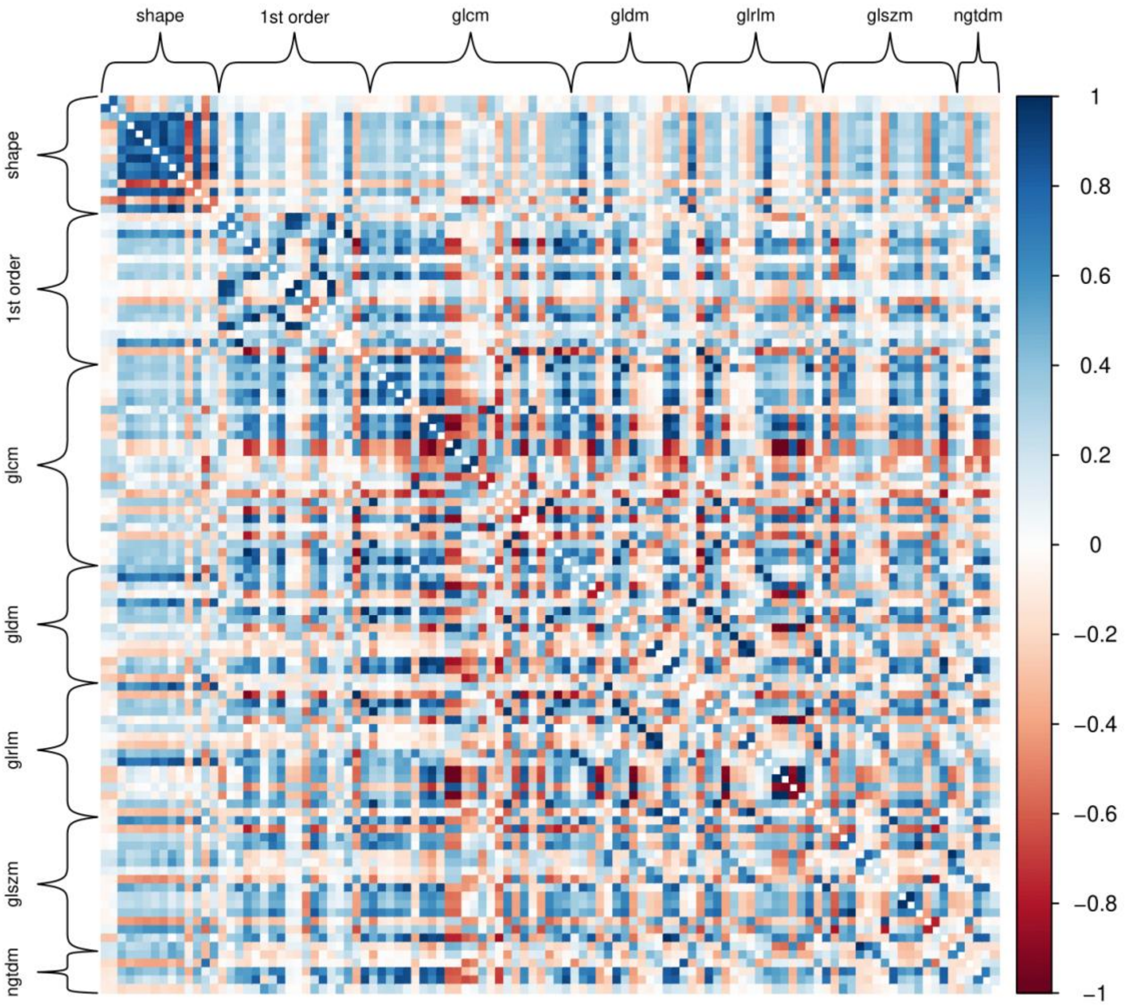

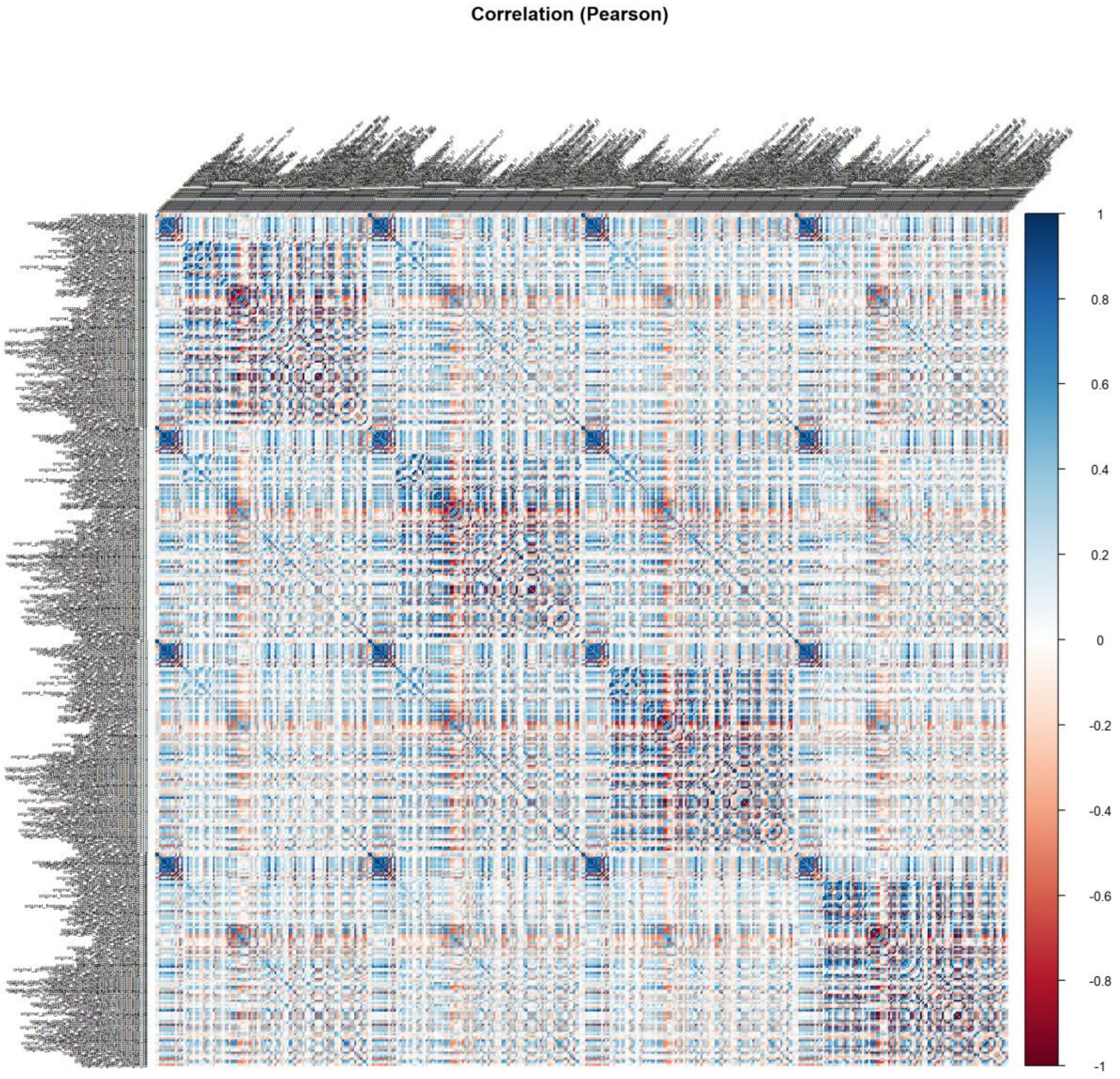

Supplementary Figure 6: Pearson correlation matrix of radiomic data of all 4 included MRI modalities (T1, T1 with contrast, FLAIR, T2).

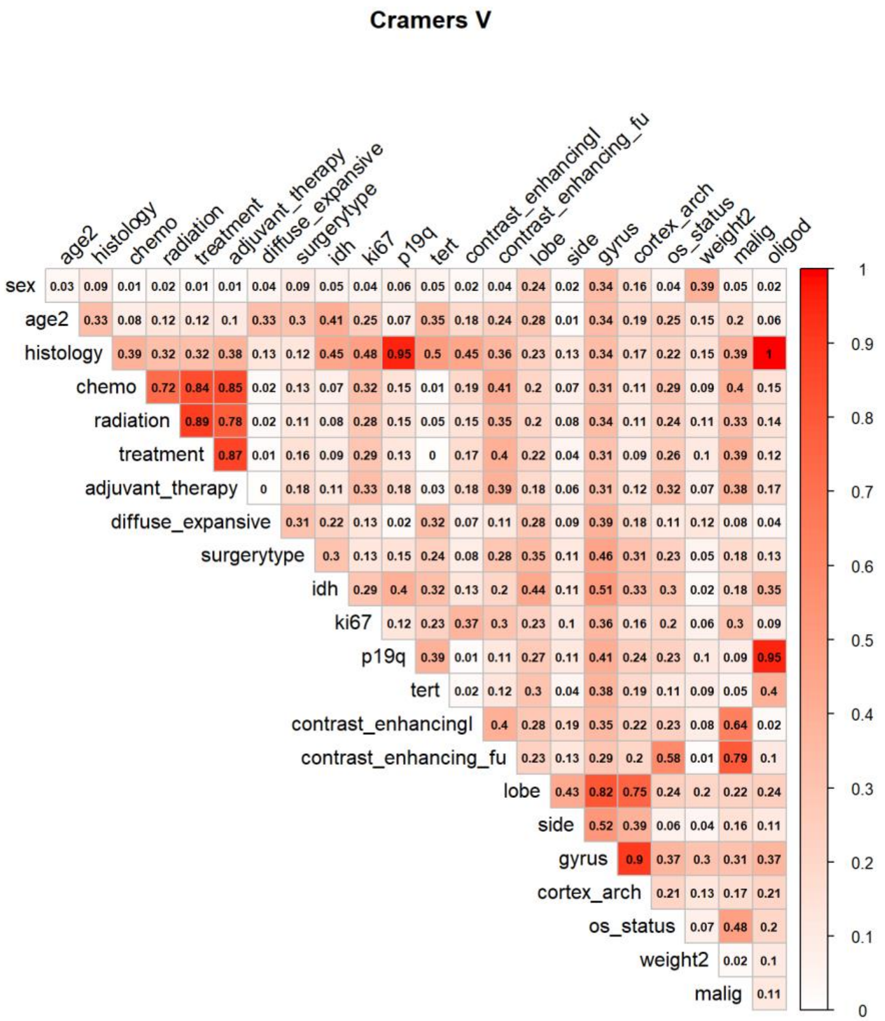

Supplementary Figure 7: Correlation matrix of clinical variables. Values range from 0 (no association between the variables) to 1 (complete association).

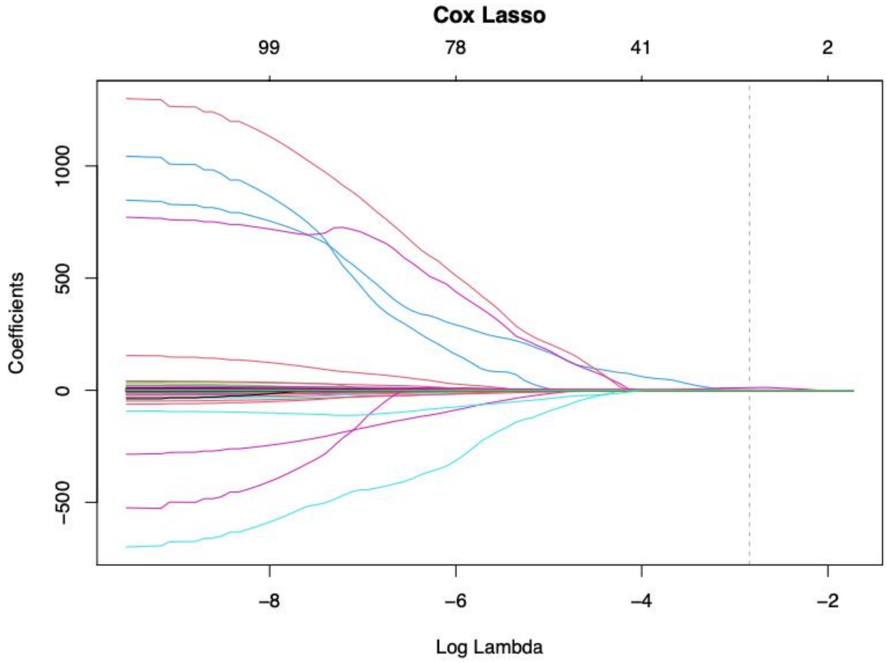

Supplementary Figure 8: Cox lasso shrinkage of coefficients towards 0. To determine the optimal value for the regularization parameter 20-fold crossvalidation (using function cv.glmnet in R-package glmnet) was performed on 10 random splittings of the data and the value yielding the smallest mean cross-validation error averaged over all splittings was chosen. The Cox lasso model was fit using the function ‘glmnet()’ withparameters alpha=1, family=’cox’ and the optimal value of λ.

Supplementary Tables

	Variable Name	Description
Patient	patient_number	Patient ID
	sex	gender (female, male)
	age	age at diagnosis
	date_diagnosis	date of diagnosis
	date_surgery	date of surgery
Treatment	chemo	chemotherapy as treatment (Yes, No)
	radiation	radiation as treatment (Yes, No)
Overall survival	os_status	Overall survival status (alive, dead, unknown)
	dod	date of death
	last_followup	date of last followup
Tumor	histology	type of tumor (Astrocytoma, Oligodendroglioma, Anplastic Astrocytoma, Anplastic Oligodendrogliom, Gemistozytic Astrocytoma)
	side	side of the tumor (right, median, left)
	surgery_type	type of surgery (Resection, Biopsy, Subtotal Resection)
	malignancy	tumor malignancy observed within observation time (Yes, No)
	date_malignancy	date of malignancy
	contrast_enhancing_I	contrast enhancing initial (Yes, No)
	contrast_enhancing_fu	contrast enhancing Followup (Yes, No)
	cortex_arch	cortex architecture (Isocortex, Mesocortex, Allocortex, Cerebellar, Multifokal, White Matter, Gliomatose)
	diffuse/expansive	diffuse or expansive tumor growth (diffuse, expansive)
Mutation, markers	idh	IDH mutation (Yes, No)
	tert	TERT mutation (wild type, mutated)
	p19q	1p/19q co-deletion: deletion of 1p and 19q chromosome arms (Yes, No)
	ki67	marker for tumor growth rate (low: 0-5%, medium: 5-10%, high: ≥ 10%)

Supplementary Table 1: Overview of clinical variables relevant for Cox-PH Models with their respective specifications.

Cox- Proportional Hazard Models - Overview

(a) Preoperative model with clinical data

	$\hat{\beta}$	$\exp(\hat{\beta})$	$se(\hat{\beta})$	DF	p-value
pspline(age.atSurg45), li	0.0446	1.0456	0.0073	1.0000	8.2e-10*
pspline(age.atSurg45), no				3.0913	0.0029*
diffuse.expansiveExpansive (ref=Diffuse)	-0.3147	0.7300	0.2180	1.0000	0.1489
cortex.archMesocortex (ref=Isocortex)	0.4199	1.5219	0.2918	1.0000	0.1501
cortex.archAllocortex (ref=Isocortex)	1.0224	2.7797	0.4263	1.0000	0.0165*
cortex.archMultifokal (ref=Isocortex)	1.3525	3.8669	0.4667	1.0000	0.0038*
cortex.archWhite Matter (ref=Isocortex)	0.8293	2.2917	0.3634	1.0000	0.0225*
cortex.archGliomatose (ref=Isocortex)	1.6740	5.3333	0.4357	1.0000	0.0001*
init.statusEnhY/MaliN (ref=EnhN/MaliN)	0.9211	2.5121	0.3071	1.0000	0.0027*
init.statusEnhY/MaliY (ref=EnhN/MaliN)	0.6647	1.9440	0.2375	1.0000	0.0051*

Supplementary Table 2: Time to death: Full Cox model using pre-op clinical data. The final model, generated by an AIC step, is summarized in table here. The results indicate noteworthy linear and non-linear effects for age. Every increase of its value by one single year enlarges the hazard by 1.0451 each. In addition, cortex.arch strongly influences the time to death. This is especially apparent for the level Gliomatose, whose hazard rate is 5.333 times higher than for the reference level Isocortex. Full Cox models include coefficient estimates $\hat{\beta}$, their standard errors $se(\hat{\beta})$, the ratio of hazard rates $\exp(\hat{\beta})$ and p-values. Also, the ratios of hazard rates $\exp(\hat{\beta})$ are displayed, which give the factor by which the hazard of an event changes compared to the reference value for a covariate. For the purpose of full disclosure, it should be remarked that z-score standardization (subtraction of the mean and division with the standard deviation) of the radiomics features was considered during data-preprocessing. However, after the Cox Lasso and AIC steps, the rerun analysis with modified features produced the same set of residual features. Also, their p-values remained unchanged.

(b) Preoperative model with clinical data including radiomic signature

	$\hat{\beta}$	exp($\hat{\beta}$)	se($\hat{\beta}$)	DF	p-value
pspline(age.atSurg45), li	0.0348	1.0354	0.0084	1.0000	3.3e-05*
pspline(age.atSurg45), no				3.0671	0.0147*
diffuse.expansiveExpansive (ref=Diffuse)	-0.0554	0.9461	0.2569	1.0000	0.8293
cortex.archMesocortex (ref=Isocortex)	0.3674	1.4439	0.3287	1.0000	0.2637
cortex.archAllocortex (ref=Isocortex)	0.7975	2.2200	0.4725	1.0000	0.0914
cortex.archMultifokal (ref=Isocortex)	1.5483	4.7036	0.4963	1.0000	0.0018*
cortex.archWhite Matter (ref=Isocortex)	0.6972	2.0082	0.3861	1.0000	0.0710
cortex.archGliomatose (ref=Isocortex)	1.0149	2.7592	0.5462	1.0000	0.0631
init.statusEnhY/MaliN (ref=EnhN/MaliN)	0.8768	2.4031	0.3163	1.0000	0.0056*
init.statusEnhY/MaliY (ref=EnhN/MaliN)	0.3536	1.4242	0.2647	1.0000	0.1817
firstorder.kurtosis.FLAIR	0.0670	1.0693	0.0721	1.0000	0.3528
gldm.dependenceentropy.T1	-0.1156	0.8908	0.3518	1.0000	0.7424
glrlm.longrunlowgraylevelemphasis.T1	4.6114	100.6216	2.3694	1.0000	0.0516
ngtdm.strength.T1	0.1071	1.1131	0.0450	1.0000	0.0172*
firstorder.mean.T1c	0.5943	1.8118	0.3049	1.0000	0.0513
firstorder.skewness.T1c	0.0525	1.0539	0.0848	1.0000	0.5361
firstorder.median.T2	-0.6617	0.5160	0.3552	1.0000	0.0625
gldm.graylevelnonuniformity.T2	0.0002	1.0002	0.0001	1.0000	0.0131*

Supplementary Table 3: Time to death: Full Cox model using radiomics and pre-op clinical data. This model is essentially a combination of models (ii) and (iv). The fusion creates a full model, while additional AIC applications produce a final model.

(c) Postoperative model including postoperative information

	$\hat{\beta}$	exp($\hat{\beta}$)	se($\hat{\beta}$)	DF	p-value
pspline(age.atSurg45), li	0.0393	1.0401	0.0092	1.0000	2.0e-05*
pspline(age.atSurg45), no				3.0567	0.4953
init.statusEnhY/MaliN (ref=EnhN/MaliN)	0.5042	1.6556	0.3452	1.0000	0.1442
init.statusEnhY/MaliY (ref=EnhN/MaliN)	0.6144	1.8486	0.2959	1.0000	0.0379*
surgerytypeBiopsy (ref=Resection)	0.9556	2.6003	0.2836	1.0000	0.0008*
surgerytypeSubtotal Resection (ref=Resection)	0.2010	1.2226	0.2862	1.0000	0.4825
histologyAstrocytoma (ref=Oligodendroglioma)	1.1405	3.1284	0.4075	1.0000	0.0051*
histologyAnplastic Astrocytoma (ref=Oligodendroglioma)	0.6996	2.0130	0.4754	1.0000	0.1411
histologyAnplastic Oligodendrogliom (ref=Oligodendroglioma)	-0.2628	0.7689	0.5621	1.0000	0.6402
histologyGemistozytic Astrocytoma (ref=Oligodendroglioma)	1.8466	6.3382	0.6589	1.0000	0.0051*
idhNo (ref=Yes)	0.8063	2.2396	0.3135	1.0000	0.0101*
ki67Medium (ref=Low)	0.7445	2.1055	0.3642	1.0000	0.0409*
ki67High (ref=Low)	1.4328	4.1903	0.4289	1.0000	0.0008* \

Supplementary Table 4: Final Cox model using post-op clinical data. The post-op clinical data adds further information to the pre-op data, including surgerytype, histology, IDH and ki67. As seen in the chart, these additional features are implied to be of great value due to strong significance in their p-values. This statement is supported by the fact that the AIC step mainly removes pre-op information like diffuse.expansive, cortex.arch or side from the model.

(d) Postoperative with all clinical data with stratification for IDH mutation

	$\hat{\beta}$	exp($\hat{\beta}$)	se($\hat{\beta}$)	DF	p-value
pspline(age.atSurg45), li	0.0348	1.0354	0.0093	1.0000	0.0002*
pspline(age.atSurg45), no				3.0689	0.3407
sideMedian (ref=Right)	0.3976	1.4882	0.4487	1.0000	0.3756
sideLeft (ref=Right)	0.5590	1.7490	0.2625	1.0000	0.0332*
init.statusEnhY/MaliN (ref=EnhN/MaliN)	0.4636	1.5898	0.3615	1.0000	0.1997
init.statusEnhY/MaliY (ref=EnhN/MaliN)	0.6869	1.9875	0.3134	1.0000	0.0284*
surgerytypeBiopsy (ref=Resection)	0.9820	2.6697	0.2912	1.0000	0.0007*
surgerytypeSubtotal Resection (ref=Resection)	0.1966	1.2172	0.2931	1.0000	0.5025
histologyAstrocytoma (ref=Oligodendroglioma)	1.3063	3.6924	0.4180	1.0000	0.0018*
histologyAnplastic Astrocytoma (ref=Oligodendroglioma)	0.5939	1.8110	0.4855	1.0000	0.2213
histologyAnplastic Oligodendrogliom (ref=Oligodendroglioma)	-0.1823	0.8333	0.5644	1.0000	0.7466
histologyGemistozytic Astrocytoma (ref=Oligodendroglioma)	1.8969	6.6655	0.6694	1.0000	0.0046*
ki67Medium (ref=Low)	0.7435	2.1033	0.3679	1.0000	0.0433*
ki67High (ref=Low)	1.5697	4.8053	0.4525	1.0000	0.0005*

Supplementary Table 5: Final Cox model using post-op clinical data with IDH stratification. Due to its significance, stratification by IDH mutation was taken into consideration. The violation of the PH assumption for this variate supported this approach. Therefore, the post-op clinical model in Section was refitted with IDH strata. Comparing the estimates and p-values to the ones from the non-stratified model unveiled only marginal differences.

(e) Postoperative model with all available clinical data including radiomic signature

	$\hat{\beta}$	exp($\hat{\beta}$)	se($\hat{\beta}$)	DF	p-value
pspline(age.atSurg45), li	0.0402	1.0410	0.0093	1.0000	1.5e-05*
pspline(age.atSurg45), no				3.0747	0.6350
surgerytypeBiopsy (ref=Resection)	0.9332	2.5425	0.2882	1.0000	0.0012*
surgerytypeSubtotal Resection (ref=Resection)	0.0845	1.0882	0.2883	1.0000	0.7694
histologyAstrocytoma (ref=Oligodendroglioma)	0.9345	2.5460	0.4050	1.0000	0.0210*
histologyAnplastic Astrocytoma (ref=Oligodendroglioma)	1.0085	2.7415	0.4505	1.0000	0.0252*
histologyAnplastic Oligodendrogliom (ref=Oligodendroglioma)	-0.1344	0.8742	0.5595	1.0000	0.8102
histologyGemistozytic Astrocytoma (ref=Oligodendroglioma)	1.3817	3.9818	0.6810	1.0000	0.0425*
idhNo (ref=Yes)	0.8962	2.4503	0.3272	1.0000	0.0062*
ki67Medium (ref=Low)	0.5863	1.7973	0.3643	1.0000	0.1076
ki67High (ref=Low)	1.2623	3.5335	0.4062	1.0000	0.0019*
glrlm.longrunlowgraylevelemphasis.T1	3.6513	38.5262	2.1130	1.0000	0.0840
ngtdm.strength.T1	0.1400	1.1503	0.0753	1.0000	0.0631
gldm.graylevelnonuniformity.T2	0.0003	1.0003	0.0001	1.0000	1.7e-05*

Supplementary Table 6: Time to death: Final Cox model using Radiomics and post-op clinical data. With the exception of init.status, every post-op clinical feature remained in the final model. Another factor with high significance was age (linear effect), surgery type, histology, idh, and ki67. When radiomics and clinical data are combined, three of the eight features are relevant enough to pass through the AIC step. To be more specific, the T1 features glrlm.longrunlowgraylevelemphasis and ngtdm.strength, as well as the T2 feature gldm.graylevelnonuniformity, remained in the final model. The latter is implied to be particularly important in terms of time to death.

(f) Radiomic model (without clinical data)

	$\hat{\beta}$	$\exp(\hat{\beta})$	$se(\hat{\beta})$	p-value
firstorder.kurtosis.FLAIR	0.1693	1.1845	0.0655	0.0097*
gldm.dependenceentropy.T1	-0.4876	0.6141	0.3102	0.1159
glrlm.longrunlowgraylevelemphasis.T1	5.9180	371.6573	2.2739	0.0093*
ngtdm.strength.T1	0.0851	1.0888	0.0391	0.0296*
firstorder.mean.T1c	0.9030	2.4671	0.2766	0.0011*
firstorder.skewness.T1c	0.1344	1.1439	0.0802	0.0936
firstorder.median.T2	-1.0861	0.3375	0.2755	8.1e-05*
gldm.graylevelnonuniformity.T2	0.0003	1.0003	0.0001	6.4e-07*

Supplementary Table 7: Time to death: Final Cox model using Radiomics data. Six of the eight attributes of the model have a significant influence on the time to death. Notable is also the fact that the final model consists solely of features based on gray levels that represent tumor heterogeneity or gray level distribution. Form based attributes are omitted from the final model.

Feature	Image	$\hat{\beta}$	Feature description	increasing hazard of death if feature value...
Firstorder kurtosis	FLAIR	0.1693*	kurtosis of gray level distribution	increases; i.e. bigger distribution tails
Firstorder mean	T1c	0.9030*	average gray level	increases; i.e. brighter gray level
Firstorder median	T2	-1.0861*	median gray level	decreases; i.e. darker gray level
Firstorder skewness	T1c	0.1344	asymmetry of gray level distribution about the mean	increases; i.e. more positive skew
GLDM graylevelnonuniformity	T2	0.0003*	similarity of gray levels	increases; i.e. bigger dissimilarity
GLDM dependence entropy	T1	-0.4876	entropy (randomness) in dependence size	decreases; i.e. smaller entropy dependence

NGTDM strength	T1	0.0851*	measure of the image primitives (contours, shapes, ...)	increases; i.e. easily visible primitives (slow change but large coarse differences in gray levels)
GLRLM longrunlowgraylevel emphasis	T1	5.9180*	measures joint dist. of long runs and low gray levels	increases, i.e. longer runs and darker gray level

Supplementary Table 8: Description and interpretation of radiomics features in final model

Time to malignancy

Event	Malignancy at diagnosis (Case 1)	Malignancy between diagnosis and surgery (Case 2)	Malignancy after surgery (Case 3)
Model	Logit	Cox PH	
Analysis of effects on ...	malignancy occurance at diagnosis	time to malignancy	
Censoring	-	interval, right	
Right-censored if ...	-	no event or event after surgery	no event
Survival time: starting point	-	date of diagnosis	date of surgery
Survival time: lower ending point	-	date of last non-malignant MRI; if right-censored: date of surgery	date of last non-malignant MRI; if right-censored: date of last follow-up
Survival time: upper ending point	-	date of malignant MRI; if right-censored: ∞	
Used information	pre-op clinical data		pre-op and post-op clinical data, radiomics
Used patients	all	non-malignancy at diagnosis	non-malignancy at or before surgery

Supplementary Table 9: Implementation details for time to malignancy. The presence of interval-censoring necessitated distinct R implementations in this instance. Cox models for interval-censored data were fitted using the R package 'icenReg'. The method 'ic sp()' with parameters model='ph' and bs samples=1000 fitted cox PH models for interval-censored data using 1000 bootstrap samples to estimate standard errors.

	$\hat{\beta}$	se($\hat{\beta}$)	p-value
sexMale (ref=Female)	-0.0991	0.2549	0.6975
diffuse.expansiveExpansive (ref=Diffuse)	0.1384	0.2714	0.6100
cortex.archNon-Isocortex (ref=Isocortex)	0.1550	0.2625	0.5547
sideLeft or Median (ref=Right)	0.6544	0.2539	0.0100*
age.atDiag45	0.0188	0.0084	0.0259*

Supplementary Table 10: Occurrence of malignancy (Case 1: at diagnosis): Logit model using pre-op clinical data

	$\hat{\beta}$	exp($\hat{\beta}$)	se($\hat{\beta}$)	p-value
sexMale (ref=Female)	0.3875	1.4730	0.3940	0.3253
diffuse.expansiveExpansive (ref=Diffuse)	-0.9050	0.4045	0.3935	0.0215*
cortex.archNon-Isocortex (ref=Isocortex)	0.0074	1.0070	0.3847	0.9847
sideLeft or Median (ref=Right)	0.3117	1.3660	0.3613	0.3884
age.atDiag45	0.0228	1.0230	0.0110	0.0378*

Supplementary Table 11: Time to malignancy (Case 2: after diagnosis, before surgery): Cox model using pre-op clinical data

	$\hat{\beta}$	exp($\hat{\beta}$)	se($\hat{\beta}$)	p-value
sexMale (ref=Female)	-0.0817	0.9215	0.3016	0.7863
diffuse.expansiveExpansive (ref=Diffuse)	-0.1918	0.8255	0.3316	0.5630
cortex.archNon-Isocortex (ref=Isocortex)	0.5794	1.7850	0.3104	0.0619
sideLeft or Median (ref=Right)	0.2185	1.2440	0.2904	0.4517
age.atSurg45	0.0059	1.0060	0.0114	0.6042

Supplementary Table 12: Time to malignancy (Case 3: after surgery): Cox model using pre-op clinical data

	$\hat{\beta}$	exp($\hat{\beta}$)	se($\hat{\beta}$)	p-value
diffuse.expansiveExpansive (ref=Diffuse)	-0.4514	0.6368	0.5061	0.3725
surgerytypeBiopsy (ref=Resection)	0.6096	1.8400	0.6539	0.3512
surgerytypeSubtotal Resection (ref=Resection)	0.2589	1.2950	0.4579	0.5718
histologyAstrocytoma (ref=Oligodendroglioma)	0.9408	2.5620	0.6373	0.1399
histologyAnplastic Astrocytoma (ref=Oligodendroglioma)	1.4550	4.2860	0.7283	0.0457*
histologyAnplastic Oligodendrogliom (ref=Oligodendroglioma)	0.3141	1.3690	4.1950	0.9403
histologyGemistozytic Astrocytoma (ref=Oligodendroglioma)	2.1290	8.4100	7.8790	0.7869
idhNo (ref=Yes)	0.5637	1.7570	0.5070	0.2662
ki67Medium (ref=Low)	-0.0634	0.9386	0.8076	0.9375
ki67High (ref=Low)	1.4290	4.1740	0.7279	0.0497*
age.atSurg45	-0.0031	0.9970	0.0169	0.8564

Supplementary Table 13: Time to malignancy (Case 3: after surgery): Cox model using post-op clinical data

	$\hat{\beta}$	exp($\hat{\beta}$)	se($\hat{\beta}$)	p-value
firstorder.kurtosis.FLAIR	0.1583	1.1710	0.1470	0.2817
gldm.dependenceentropy.T1	0.3017	1.3520	0.5691	0.5960
glrlm.longrunlowgraylevelemphasis.T1	5.4860	241.2000	11.9600	0.6466
ngtdm.strength.T1	-0.0675	0.9347	0.3856	0.8610
firstorder.mean.T1c	0.5436	1.7220	0.5461	0.3195
firstorder.skewness.T1c	0.0398	1.0410	0.1708	0.8155
firstorder.median.T2	-0.6722	0.5106	0.5087	0.1864
gldm.graylevelnonuniformity.T2	0.0003	1.0000	0.0002	0.0245*

Supplementary Table 14: Time to malignancy (Case 3: after surgery): Cox model using Radiomics data

	$\hat{\beta}$	$\exp(\hat{\beta})$	$se(\hat{\beta})$	p-value
firstorder.kurtosis.FLAIR	0.0098	1.0100	0.2179	0.9640
gldm.dependenceentropy.T1	-0.1448	0.8652	1.0400	0.8893
glrlm.longrunlowgraylevelemphasis.T1	12.8200	370500.0000	18.3200	0.4839
ngtdm.strength.T1	-0.0608	0.9410	0.5576	0.9132
firstorder.mean.T1c	0.4157	1.5150	0.8390	0.6203
firstorder.skewness.T1c	-0.0692	0.9331	0.2810	0.8054
firstorder.median.T2	-0.7249	0.4844	0.9229	0.4322
gldm.graylevelnonuniformity.T2	0.0003	1.0000	0.0003	0.2233
diffuse.expansiveExpansive (ref=Diffuse)	0.0726	1.0750	0.7505	0.9229
surgerytypeBiopsy (ref=Resection)	0.4663	1.5940	0.9315	0.6167
surgerytypeSubtotal Resection (ref=Resection)	0.3540	1.4250	0.5960	0.5526
histologyAstrocytoma (ref=Oligodendroglioma)	0.9779	2.6590	0.8663	0.2590
histologyAnplastic Astrocytoma (ref=Oligodendroglioma)	1.7200	5.5820	1.0910	0.1149
histologyAnplastic Oligodendrogliom (ref=Oligodendroglioma)	0.3008	1.3510	3.6720	0.9347
histologyGemistozytic Astrocytoma (ref=Oligodendroglioma)	1.8480	6.3470	7.2140	0.7978
idhNo (ref=Yes)	0.5499	1.7330	0.7105	0.4390
ki67Medium (ref=Low)	-0.5430	0.5810	0.9620	0.5725
ki67High (ref=Low)	1.0540	2.8690	0.9595	0.2720
age.atSurg45	-0.0079	0.9921	0.0211	0.7075

Supplementary Table 15: Time to malignancy (Case 3: after surgery): Cox model using Radiomics and post-op clinical data

	Estimate	Std. Error	t value	Pr(>|t|)
(Intercept)	8.39562	11.29457	0.743	0.459
ki67Medium	0.07359	3.6278	0.02	0.984
ki67High	0.53268	6.10489	0.087	0.931
p19qYes	-2.83886	10.84047	-0.262	0.794
histologyAstrocytoma	-0.11061	10.88341	-0.01	0.992
histologyAnplastic Astrocytoma	14.86167	12.16089	1.222	0.224
histologyAnplastic Oligodendrogliom	14.79692	18.61609	0.795	0.428
histologyGemistozytic Astrocytoma	10.52207	11.4823	0.916	0.361
idhNo	1.51075	4.52924	0.334	0.739
age_atDiag2>= 45Y	5.84794	3.65134	1.602	0.112
diffuse_expansiveExpansive	-2.0872	3.59462	-0.581	0.563
sideLeft	-0.22507	3.26255	-0.069	0.945

Supplementary Table S16: Linear model for velocity of diameter expansion per year. Cortex architecture was not considered due to sparsity.

	N	Min.	1st Qu.	Median	Mean	3rd Qu.	Max.
sex: Female	55	-2.98	3.04	4.56	9.56	9.69	134.68
sex: Male	85	-0.87	3.11	5.56	10.66	10.28	112.53
age at Diagnosis: < 45 years	96	-2.98	3.09	5.09	7.57	9.28	59.21
age at Diagnosis: >= 45 years	44	-0.19	3.04	5.19	16.02	15.95	134.68
histology: Oligodendroglioma	52	-0.87	2.92	4.51	6.13	6.9	48.97
histology: Astrocytoma	64	-2.98	2.96	5.04	8.94	10.29	61.63
histology: Anplastic Astrocytoma	15	1.05	3.3	11.05	26.01	22.52	134.68
histology: Anplastic Oligodendrogliom	2	5.08	8.46	11.84	11.84	15.22	18.6
histology: Gemistozytic Astrocytoma	7	3.62	6.14	9.34	18.13	21.23	59.21
side: Right	75	-2.98	3.16	5.08	10.24	10.47	112.53
side: Median	0						
side: Left	65	-0.87	3	5.24	10.2	9.73	134.68
Cortex arch: Isocortex	101	-2.98	3.2	5.43	11.34	9.93	134.68
Cortex arch: Mesocortex	22	-0.19	2.26	3.3	4.63	5.45	15.93
Cortex arch: Archicortex	8	-0.43	2.64	9.5	10.73	15.76	31.26
Cortex arch: Multifokal	2	1.81	2.62	3.44	3.44	4.26	5.08
Cortex arch: White Matter	6	4.17	4.89	8.44	9.73	14.58	17.01
Cortex arch: Gliomatose	1	32.72	32.72	32.72	32.72	32.72	32.72
diffuse/expansive: Diffuse	53	-0.43	3.11	5.08	12.72	11.05	112.53
diffuse/expansive: Expansive	87	-2.98	3.04	5.24	8.7	9.49	134.68
contrast enhancing init: no	95	-2.98	2.98	4.79	8.75	9.45	112.53
contrast enhancing init: yes	45	-0.87	3.2	6.34	13.34	12.38	134.68
contrast enhancing fu: no	71	-2.98	2.5	4.45	6.08	7.91	31.26
contrast enhancing fu fu: yes	69	-0.87	3.53	5.54	14.48	15.28	134.68
idh: no	27	-0.05	3.84	6.39	17.96	20.42	112.53
idh: yes	116	-2.98	2.95	5.1	8.66	9.26	134.68
p19q: no	87	-2.98	3	5.17	12.7	11.49	134.68
p19q: yes	5	-0.87	2.97	4.98	6.57	7.11	48.97
ki67: Low	86	-0.43	3.04	4.56	8.76	8.28	134.68
ki67: Medium	49	-2.98	2.36	5.48	9.72	14.68	48.97
ki67: High	27	2.4	5.45	9.99	18.45	17.41	112.53

Supplementary Table 17: Descriptives of velocity diameter expansion per year in subgroups defined by clinical parameters.

Coefficients:				
	Estimate	Std. Error	t value	Pr>t]
(Intercept)	1.0067313	0.0013749	732.228	< 2e-16 ***
original_glszm_largearealowgraylevelemphasis_FLAIR	0.004141	0.0014812	2.796	0.005652 **
original_gldm_dependencevariance_T1	0.0010998	0.0020761	0.53	0.596841
original_firstorder_mean_Tic	0.0015466	0.0014594	1.06	0.290453
original_glcm_clustershade_Tic	0.0044726	0.0012241	3.654	0.000325
original_gldm_dependencevariance_Tic	0.0027684	0.0021272	1.301	0.194513
original_glszm_largeareaemphasis_Tic	-0.0036044	0.0014245	-2.53	0.012120 *
original_firstorder_kurtosis_T2	0.0023212	0.0012471	1.861	0.064087 .
original_firstorder_maximum_T2	0.0025688	0.0017186	1.495	0.136476
original_firstorder_skewness_T2	-0.0005929	0.0019338	-0.307	0.759462
original_glcm_clustershade_T2	0.0029916	0.0016257	1.84	0.067137 .
original_gldm_largedependencelowgraylevelemphasis_T2	0.0011518	0.0018902	0.609	0.54294
original_glszm_largearealowgraylevelemphasis_T2	0.0005427	0.0015262	0.356	0.722476
original_ngtdm_strength_T2	0.0037104	0.0015865	2.339	0.020280 *

Supplementary table 18: Linear model for growth rate per day based on LASSO selected radiomic parameters.

Variables		Code
sex	Sex	1=female, 2=male
previous_allocation	Previous Allocation	1=Private Household, 2=Home for the elderly, 3=Otherwise allocated, 4=Different Hospital, 5=Own Hospital / Different Department, 6=Nursing Home, 7=Psychiatric Clinic, 8=Rehabilitation Clinic, 9=At Home with Professional Help
behavior	Patient Behavior	1=Normal, 2=Conspicuous
smoker	Patient smokes	0=N/A, 1=No never, 2=No stopped, 3=Yes
alcohol_abuse	Alcohol Abuse	0=no, 1=yes
heart		0=no, 1=yes
vessels		0=no, 1=yes
hematology		0=no, 1=yes
lung		0=no, 1=yes
liver		0=no, 1=yes
kidneys		0=no, 1=yes
metabolic		0=no, 1=yes
allergies		0=no, 1=yes
nervous_system		0=no, 1=yes
gastrointestinal		0=no, 1=yes
skeletal		0=no, 1=yes
muscular		0=no, 1=yes
adjuvant_therapy		0=no, 1=yes
chemo		0=no, 1=yes
chemo2		0=no, 1=yes
chemo3		0=no, 1=yes
radiation		0=no, 1=yes
radiation2		0=no, 1=yes
radiation3		0=no, 1=yes
secondline_chemo	Secondline Chemotherapy	0=N/A, 1=Avastin, 2=CCNU
surgerytype	Type of Surgery	1=Resection, 2=Biopsy, 3=Subtotal Resection
surgery_date		
pet_positive	PET positive	0=no, 1=yes
multifocal		0=no, 1=yes
contrast_enhancingI	Contrast Enhancing Initial	0=no, 1=yes
contrast_enhancing_fu	Contrast Enhancing Followup	0=no, 1=yes
dom_rad_available	Date of Malignancy (Radiol.) available	0=no, 1=yes
dom_hist_available	Date of Malignancy (Hist.) available	0=no, 1=yes
infra_supratentorial	Infra-/Supratentorial	1=Infra, 2=Supra, 3=Infra/Supra
diffuse_expansive	Diffuse/Expansive	1=Diffuse, 2=Expansive
loc_intraparanchymal	Location Intraparenchymal	1=Hemispherical, 2=Cerebellar, 3=Mesencephalon, 4=brain stem, 5=Insula, 6=Thalamus, 7=Multifokal, 8=gliomatosis
lobe		1=Frontal, 2=Temporal, 3=Occipital, 4=Parietal, 5=Central Region, 6=Corpus Callosum, 7=Cerebellar, 8=Hypothalamus, 9=Insula, 10=Thalamus, 11=Multifokal, 12=Mesencephalon, 13=Pons, 14=gliomatosis
side		1=right, 2=median/bilateral, 3=left
gyrus		1=Angularis, 2=Cerebellar, 3=Cingulum, 4=Corona Radiata, 5=Cuneus, 6=F1, 7=F2, 8=F3, 9=Fusiform, 10=Gyrus rectus, 11=Hypothalamus, 12=InsularTrunc (short gyri), 13=Insula, 14=Lobulus Paracentralis, 15=Lobulus Parietalis Superior, 16=Medulla, 17=Multifokal, 18=O1, 19=O2, 20=O3, 21=Orbitofrontal
cortex_arch		1=Isocortex, 2=Mesocortex, 3=Allocortex, 4=Cerebellar, 5=Multifokal, 6=WM, 7=gliomatosis
ala	5-ALA	0=no, 1=yes
intraoperative_mri	Intraoperative MRI	0=no, 1=yes

histology		1=Astrocytoma, 2=Oligodendroglioma, 3=Anaplastic Astrocytoma, 4=Anplastic Oligodendroglioma, 5=Glioblastoma, 6=Gemistozytic Astrocytoma, 7=Oligoastrocytoma
p19q	1p19q	0=no, 1=yes
mgmt		0=no, 1=yes
tert		0=N/A, 1=WT, 2=mutated
tert_promoter		0=N/A, 1=C228T, 2=C250T
idh		0=no, 1=yes
ki67		0=N/A, 1=0-5% low, 2=5-10% medium, 3=>10% high
epc		0=no, 1=yes
os_status	Overall Survival Status	0=N/A, 1=Alive, 2=Dead
os_available	Overall Survival available	0=no, 1=yes

Supplementary Table 19 An overview of the protocol framework for the data collected for each recruited patient. Parameters that require values or dates to be entered (for example, surgery date) are not displayed.

**MRI Scanner List**
GE MEDICAL SYSTEMS GENESIS_SIGNA (1.5T)
GE MEDICAL SYSTEMS Optima MR450w (1.5T)
GE MEDICAL SYSTEMS Signa HDxt(1.5T)
Hitachi Medical Corporation APERTO_Lucent (0.4T)
Philips Healthcare Ingenia (1.5T)
Philips Healthcare Ingenia (3T)
Philips Medical Systems Achieva (1.5T)
Philips Medical Systems Achieva (3T)
Philips Medical Systems Achieva dStream (3T)
Philips Medical Systems Gyroscan Intera (1T)
Philips Medical Systems Gyroscan Intera (1.5T)
Philips Medical Systems Gyroscan NT Intera (1.5T)
Philips Medical Systems Ingenia (1.5T)
Philips Medical Systems Ingenia (3T)
Philips Medical Systems Ingenia S (1.5T)
Philips Medical Systems Intera (1T)
Philips Medical Systems Intera (1.5T)
Philips Medical Systems Multiva (1.5T)
Philips Medical Systems NT Intera (1.5T)
Philips Medical Systems Panorama HFO (1T)
SIEMENS Aera (1.5T)
SIEMENS Avanto (1.5T)
SIEMENS Avanto_fit (1.5T)
SIEMENS Espree (1.5T)
Siemens MAGNETOM Aera (1.5T)

Siemens MAGNETOM Altea (1.5T)
SIEMENS MAGNETOM EXPERT (1T)
SIEMENS MAGNETOM EXPERT plus (1T)
SIEMENS MAGNETOM Harmony (1)
SIEMENS MAGNETOM Symphony (1.5T)
Siemens MAGNETOM Vida (3T)
SIEMENS NUMARIS/4 (1.5T)
SIEMENS Siemens Magnetom (1.5T)
SIEMENS Siemens Magnetom (3T)
SIEMENS Skyra (3T)
SIEMENS Skyra_fit (3T)
SIEMENS Sonata (1.5T)
SIEMENS Symphony (1.5T)
SIEMENS SymphonyTim (1.5T)
SIEMENS syngo.via.VB10A (3T)
SIEMENS syngo.via.VA20A (3T)
SIEMENS TrioTim (3T)
SIEMENS Verio (3T)

Supplementary Table 20: An overview of all MRI scanners used to generate MRI images for our patient cohort.

R packages

Anderson-Bergman, C. (2017). icenReg: Regression Models for Interval Censored Data in R. *Journal of Statistical Software*, Nov 2017, Vol 81(12), 1–23, doi: 10.18637/jss.v081.i12

Canty, A., Ripley, B. (2021). *boot: Bootstrap R (S-Plus) Functions*. R package version 1.3-28. URL: https://CRAN.R-project.org/package=boot

Heagerty, P. J., Saha-Chaudhuri, P. (2022). *risksetROC: Riskset ROC curve estimation from censored survival data.* R package version 1.0.4.1. URL: https://CRAN.R-project.org/package=risksetROC

Kuhn, M. (2022). *caret: Classification and Regression Training.* R package version 6.0-92. URL: https://CRAN.R-project.org/package=caret

R Core Team (2022). *R: A language and environment for statistical computing.* R Foundation for Statistical Computing, Vienna, Austria. URL: https://www.R-project.org/.

Therneau, T. (2022). *survival: A Package for Survival Analysis in R.* R package version 3.3-1, URL: https://CRAN.R-project.org/package=survival.

R code is made available online via https://github.com/PRauch1/Natural-History-LGG-R-Code.git
